# SnoRNA Evolution, Genomic Context, and Mobility: The Role of Function and Host Gene Association

**DOI:** 10.1093/gbe/evag269

**Published:** 2026-10-24

**Authors:** Samuel Coderre, Alphonse Birane Thiaw, Michelle S Scott

**Affiliations:** Département de biochimie et de génomique fonctionnelle, Faculté de médecine et des sciences de la santé, Université de Sherbrooke, Sherbrooke, Québec J1E 4K8, Canada; Centre de recherche du Centre hospitalier universitaire de Sherbrooke (CRCHUS), Sherbrooke, Québec J1H 5N3, Canada; Département de biochimie et de génomique fonctionnelle, Faculté de médecine et des sciences de la santé, Université de Sherbrooke, Sherbrooke, Québec J1E 4K8, Canada; Centre de recherche du Centre hospitalier universitaire de Sherbrooke (CRCHUS), Sherbrooke, Québec J1H 5N3, Canada; Département de biochimie et de génomique fonctionnelle, Faculté de médecine et des sciences de la santé, Université de Sherbrooke, Sherbrooke, Québec J1E 4K8, Canada; Centre de recherche du Centre hospitalier universitaire de Sherbrooke (CRCHUS), Sherbrooke, Québec J1H 5N3, Canada

**Keywords:** small nucleolar RNA (snoRNA), host gene, vertebrate evolution, gene mobility, noncoding RNA

## Abstract

Small nucleolar RNAs (snoRNAs) are best known for guiding chemical modifications of ribosomal and spliceosomal RNAs. Most vertebrate snoRNAs are encoded within introns of host genes and therefore depend on the production and processing of host precursor RNAs for their biogenesis. Despite this close genomic association, how host–gene context is associated with snoRNA abundance and evolution remains poorly understood. Here, we combined human snoRNA annotations from snoDB, Ensembl gene annotations, snoRNA-sensitive abundance data across multiple tissues, and vertebrate orthology analyses to systematically characterize snoRNA–host–gene relationships. Host genes were grouped into functional classes to compare snoRNA genomic organization, abundance, and conservation across host contexts. We confirmed that snoRNAs are enriched in ribosome-related and lncRNA host genes. SnoRNAs embedded in ribosome-related host genes, particularly ribosomal protein genes, showed the highest abundance and were predominantly broadly detected across tissues, whereas snoRNAs in other host classes were generally less abundant and more tissue-restricted. Across vertebrates, snoRNA sequences were often widely conserved, but the exact host–gene association was less stable, showing that sequence conservation can persist despite changes in genomic embedding. Ribosome-related host classes nevertheless emerged as comparatively stable environments. Together, these results show that snoRNA–host–gene relationships vary across host contexts and evolutionary histories and are consistent with a conceptual continuum from functionally concordant associations to more independent passenger relationships.

Significance Many vertebrate snoRNAs are located in introns of other genes, but this arrangement has often been treated as a feature of genome organization with little consideration of how it may influence snoRNA biology. What has been lacking is a comprehensive view of whether different host genes provide different environments for snoRNA production and long-term maintenance. We show that snoRNAs are not simply randomly distributed among host genes: some host contexts, especially genes linked to ribosome production and translation, are associated with high snoRNA expression and stronger evolutionary stability, whereas other contexts are more variable. This helps to explain why some snoRNA–host gene pairs are tightly connected while others behave more like flexible genomic arrangements, and our approach provides a framework for studying host genes as active contributors to snoRNA evolution rather than passive containers.

## Introduction

Small nucleolar RNAs (snoRNAs) are an abundant class of small noncoding RNAs that play significant roles in the maturation and modification of other RNA molecules. Their best-characterized function is to guide site-specific chemical modifications of ribosomal RNAs (rRNAs) and small nuclear RNAs (snRNAs), two RNA classes that are central to the biological processes of translation and pre-mRNA splicing (Kiss 2001; Matera et al. 2007; Kufel and Grzechnik 2019). To perform this function, snoRNAs associate with conserved proteins, forming small nucleolar ribonucleoprotein complexes (snoRNPs). Box C/D snoRNPs contain fibrillarin, NOP56, NOP58, and SNU13 and guide 2′-O-methylation, whereas box H/ACA snoRNPs contain dyskerin, NOP10, NHP2, and GAR1 and guide pseudouridylation (Kiss 2001; Filipowicz and Pogacić 2002). A specialized subset, the small Cajal body–specific RNAs (scaRNAs), localizes to Cajal bodies and mainly guides these modifications on spliceosomal snRNAs (Matera et al. 2007).

Beyond these canonical activities, some snoRNAs play other functional roles including in alternative splicing and gene-expression control, guiding cytidine acetylation, regulating tRNA homeostasis, or modulating signal recognition particle RNA (7SL) (Sharma et al. 2017; Kufel and Grzechnik 2019; Bergeron et al. 2020; Zhang et al. 2023; Liu et al. 2025). This functional diversity raises the question of whether distinct snoRNA roles are associated with particular genomic contexts and conservation patterns.

In vertebrate genomes, most snoRNAs are encoded within introns of larger transcription units referred to as host genes, whereas a smaller subset occurs in intergenic transcription units with their own promoters (Fig. 1a; Dieci et al. 2009). Early discovery efforts identified many snoRNAs housed in ribosomal protein, translation, and ribosome biogenesis genes, including genes of the 5′ terminal oligopyrimidine (5′ TOP) class whose translation is coordinated with cellular growth and metabolic state (Tycowski et al. 1996; Nepal et al. 2020). The concordance between ribosome-related host functions and canonical snoRNA targets has therefore been interpreted as functional coupling (Hoeppner and Poole 2012; Bergeron et al. 2023a). However, most human snoRNAs are encoded within genes outside protein production, including long noncoding RNAs, and the generality of this relationship remains unclear.

**Fig. 1.**
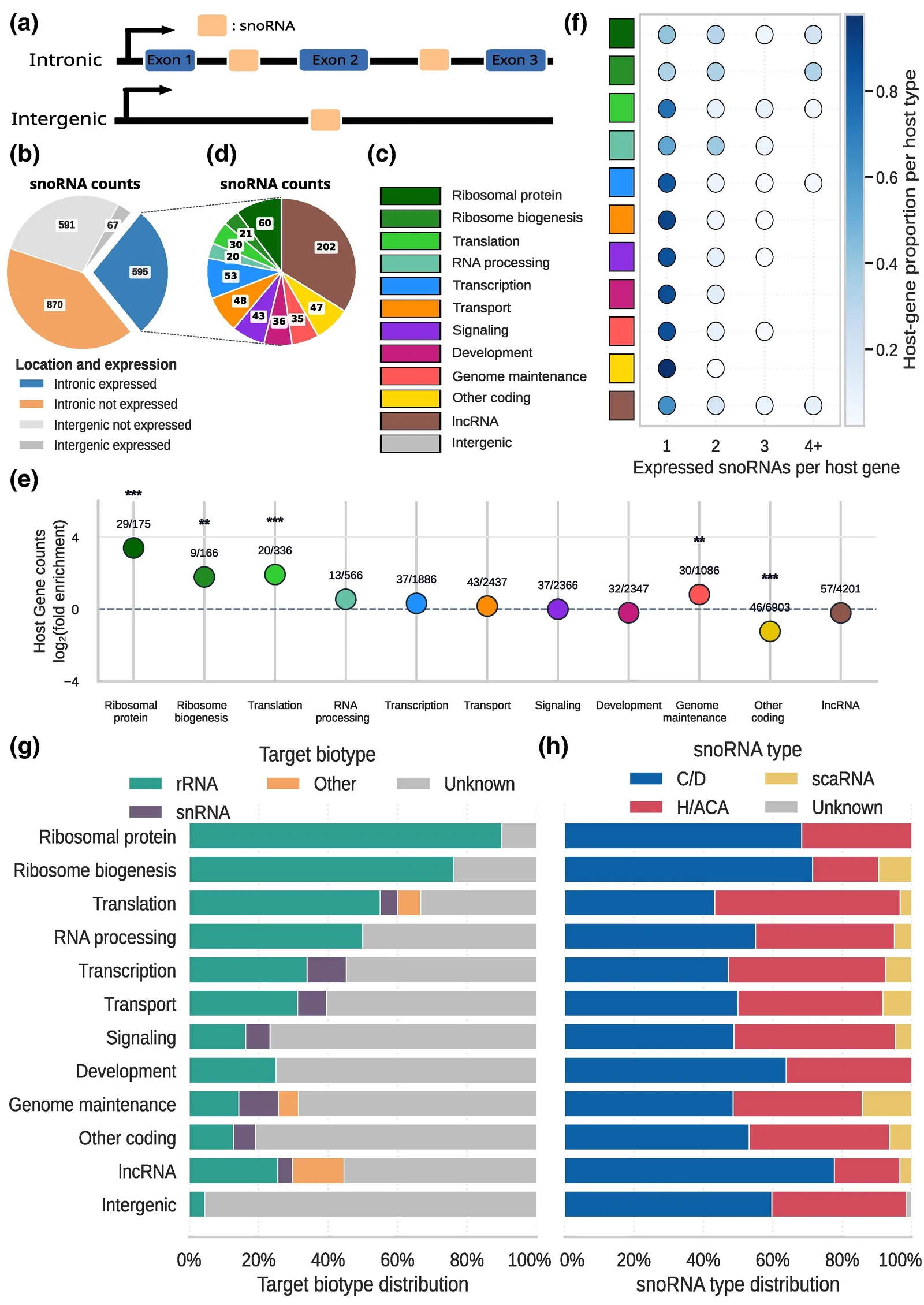
Distribution and enrichment of snoRNAs across host-gene classes. a) Schematic comparison of intronic and intergenic snoRNAs. b) Doughnut chart partitioning all 2,123 human snoRNAs into expressed intronic (*n* = 595), undetected intronic (*n* = 870), expressed intergenic (*n* = 67), and undetected intergenic (*n* = 591) categories. c) Color key and labels for the 11 operational host–gene classes and the intergenic category. d) Distribution of the 595 expressed intronic snoRNAs among host classes; counts are shown directly. e) Log_2_-transformed fold enrichment of the number of host genes belonging to each class relative to the corresponding background of all expressed genes, displayed on a linear *y*-axis from −4 to 4 with a reference line at 0, host/background gene counts, and significance from 2-sided Fisher exact tests after Benjamini–Hochberg correction (* adjusted *P* < 0.05, ** adjusted *P* < 0.01, and *** adjusted *P* < 0.001). f) Distribution of the number of expressed snoRNAs per host gene; sample sizes are shown for each class. g, h) Target-biotype and snoRNA-type distributions among expressed snoRNAs in each host class.

The intronic organization of snoRNAs also links their biogenesis to the production and splicing of the host precursor RNA. During pre-mRNA processing, core proteins protect intronic snoRNAs from exonucleolytic degradation after intron excision, making mature snoRNA production sensitive to features of the host intron (Yang 2015). For box C/D snoRNAs, processing efficiency depends in part on position relative to the intronic branch point (Hirose and Steitz 2001). Alternative routes also exist: some intronic snoRNAs can undergo endonucleolytic maturation, and SCARNA28, a scaRNA recently characterized for its unusual biogenesis strategy, can be produced through both intronic processing and an independent intronic promoter (de Turris et al. 2004; Izumikawa et al. 2025). Intron architecture may therefore be associated with mature snoRNA abundance, although it does not by itself identify the rate-limiting process.

The abundance of mature snoRNAs and host transcripts are often only modestly correlated and can reflect differences in transcription, splicing and intron processing, RNA stability, and decay (Fafard-Couture et al. 2021). Several noncoding host genes, including SNHG family members, primarily carry intronic snoRNAs; their spliced host transcripts may be degraded rapidly, including through nonsense-mediated decay, while the intron-derived snoRNAs remain stable (Tycowski et al. 1996; Lykke-Andersen et al. 2014; Bergeron et al. 2021). Alternative promoters can further change host-transcript stability or processing without equivalent changes in snoRNA maturation. Conversely, SNORD86 and SNORD2 directly regulate splicing of their own host transcripts, showing that some relationships extend beyond shared localization and functional coupling to cis-regulatory interactions (Lykke-Andersen et al. 2018; Bergeron et al. 2023a).

SnoRNA families can also expand or change genomic context through proposed processes that include duplication, loss, retrotransposition, and recombination (Weber 2006; Schmitz et al. 2008; Bergeron et al. 2021). Under the constrained-drift model of evolution of snoRNAs, sequences and genomic positions may change, but selection maintains sufficient expression and target recognition (Hoeppner and Poole 2012). In accordance with this model, higher eukaryotic genomes contain multiple copies of many snoRNA families, including copies not detected in available expression data (Bergeron et al. 2021; Fafard-Couture et al. 2023; Fafard-Couture et al. 2024). These processes provide hypotheses for snoRNA mobility, but changes in host association alone do not identify the underlying mechanism.

SnoRNAs and their host genes clearly have multiple levels of relationships that shape their evolution and often cannot be dissociated from their functions. However, while many reports cited above have provided clues about these relationships, these findings were often serendipitous or byproducts of the investigation of other research questions, and they have only been described partially and for a small number of snoRNA–host gene pairs. Here, we systematically investigate joint characteristics of snoRNAs and their host genes, benefiting from the fact that snoRNA and host gene annotations have considerably improved in humans in recent years and are now available across hundreds of vertebrates. We find that snoRNA–host gene relationships are highly structured yet heterogeneous, with host gene context being associated with distinct genomic architectures, abundance profiles, and evolutionary dynamics. We identify ribosome/translation-related host genes as forming a particularly coherent group characterized by compact genomic architecture, high snoRNA density, strong expression, and high evolutionary conservation, while other host gene classes provide more variable genomic environments, supporting a wider diversity of snoRNA expression patterns and evolutionary behaviors. Overall, our results indicate that snoRNA characteristics are associated with the genomic and functional context of their host genes.

## Results

### Genomic Organization and Host Gene Context of Human snoRNAs

We used our current dataset of human snoRNAs, housed in snoDB v2 (Bergeron et al. 2023b), which contains 2,123 human snoRNAs, the majority of which (1,465/2,123) are located within introns of host genes, the remainder being intergenic (Fig. 1b). To determine their expression status, we used low-structure-bias TGIRT-seq datasets prepared from rRNA-depleted total RNA without poly(A) selection, covering diverse normal human tissues (liver, skeletal muscle, prostate, breast, ovary, testis, colon, lung, brain, fetal brain, and placenta). SnoRNAs were labeled as expressed if they were detected above 1 transcript per million (TPM) in at least one tissue. As expected, a higher proportion of intronic snoRNAs were expressed than intergenic snoRNAs (40.6% vs. 10.2%) (Fig. 1b), consistent with observations we reported previously considering a smaller number of tissues (Fafard-Couture et al. 2021, 2023).

We next defined host gene classes of expressed snoRNAs. Genes encoding proteins with functions related to ribosome, translation regulation, and nucleolar function have long been known as snoRNA host genes (Maxwell and Fournier 1995; Dieci et al. 2009). In addition, the first snoRNA host genes identified also included some of the first long noncoding RNAs reported (Frey et al. 1997; Pelczar and Filipowicz 1998; Smith and Steitz 1998). However, as more intronic snoRNAs were identified, host genes encoding proteins with increasingly diverse cellular roles were also recognized. To define snoRNA host gene classes, we first collected all snoRNAs hosted in ribosomal protein genes. Previous studies have indicated that this group of host genes displays a different level of abundance correlation with their encoded snoRNAs, and thus, we kept this class separate (Fafard-Couture et al. 2021). We also defined lncRNAs as a separate class. For the remaining 333 host-encoded expressed snoRNAs, we retrieved biological process Gene Ontology (GO) annotations for their host genes and grouped related biological processes into broader functional classes. In total, we defined 8 additional protein-coding host gene classes, with unassigned protein-coding hosts grouped as other coding, bringing the total to 11 host gene classes overall (Fig. 1c). For genes associated with several biological processes, the category represented by the largest number of informative biological process and molecular function GO annotations was retained; ties were resolved using the first category in the predefined classification order (Fig. S1).

Expressed snoRNAs were not uniformly distributed across host gene classes but instead showed clear enrichment in specific categories. LncRNAs hosted 202 snoRNAs (33.9% of expressed host-encoded human snoRNAs), followed by ribosomal protein genes with 60 snoRNAs (10.1%); the other classes each hosted 3.4% to 8.9% of expressed snoRNAs (Fig. 1d). To compare host representation with the background of all expressed genes, we calculated fold enrichment for each host gene class (Fig. 1e). Ribosomal protein, ribosome biogenesis, translation, and genome-maintenance hosts showed fold enrichments of 10.55, 3.45, 3.79, and 1.76, respectively, compared to the background of all expressed human genes, whereas other-coding hosts were depleted (0.42-fold). Two-sided Fisher exact tests with Benjamini–Hochberg correction yielded adjusted *P*-values of 1.26 × 10^−18^, 0.00459, 3.02 × 10^−6^, 0.00832, and 8.15 × 10^−14^, for these classes, respectively; host and background gene counts are shown in Fig. 1e.

In addition, the host gene classes of ribosomal protein genes and ribosome biogenesis genes were enriched for snoRNA content. Indeed, as shown in Fig. 1f, these genes carried multiple expressed snoRNAs (4 or more snoRNAs) more often than other classes of genes (one-sided Fisher exact *P*-values = 1.69e−03 and 7.75e−03, respectively), which typically hosted only a single expressed snoRNA. This contrast was observed for 33.0% of ribosome-biogenesis hosts and 20.7% of ribosomal protein hosts, whereas most other host classes contained one expressed snoRNA. These results indicate that certain host classes represent dense multi-snoRNA loci, whereas others act primarily as single-snoRNA carriers; they do not by themselves establish that host identity causes coordinated production.

Functional differences between host gene classes were also reflected in the types and targets of the snoRNAs they harbor. SnoRNAs embedded in ribosomal protein and ribosome biogenesis genes were overwhelmingly associated with canonical substrates (rRNA and snRNA; 90.0% and 76.2%, respectively; one-sided Fisher exact *P*-values = 1.71e−19 and 1.92e−04, respectively), whereas snoRNAs without known targets (Unknown in Fig. 1g) were more common in classes not related to ribosomal protein, ribosome biogenesis, or translation genes (69.0% vs. 20.7% in ribosome- and translation-related host classes; one-sided Fisher exact *P*-value = 7.92e−21), particularly signaling, development, transport, and other coding genes (Fig. 1g). SnoRNA type proportions also varied among host classes (Fig. 1h), indicating an association between host gene context and distinct structural and functional snoRNA repertoires.

Taken together, these results show that snoRNA genomic organization is highly structured and closely linked to host gene identity. Rather than serving as passive carriers, host genes appear to define distinct genomic environments related to snoRNA coregulation and functional composition.

### Intron Architecture Differs Among snoRNA Host Gene Classes

Because intronic snoRNA maturation depends on splicing, we next examined whether the architecture of snoRNA-containing introns varies according to host gene type. Differences in intron structure could influence snoRNA processing efficiency and thereby contribute to the snoRNA-host gene pairing patterns observed above.

The distance between the 3′ end of the snoRNA and the 3′ end of the host intron differed markedly between classes (Fig. 2a). The shortest median distances were observed in ribosomal protein genes (109 nt), lncRNAs (247 nt), and translation genes (168 nt), whereas much larger distances were observed in development genes (3,911 nt) and signaling genes (3,374 nt). This proximity is consistent with known positional constraints on box C/D snoRNA maturation (Hirose and Steitz 2001), but the present analysis does not establish a difference in processing rate.

**Fig. 2.**
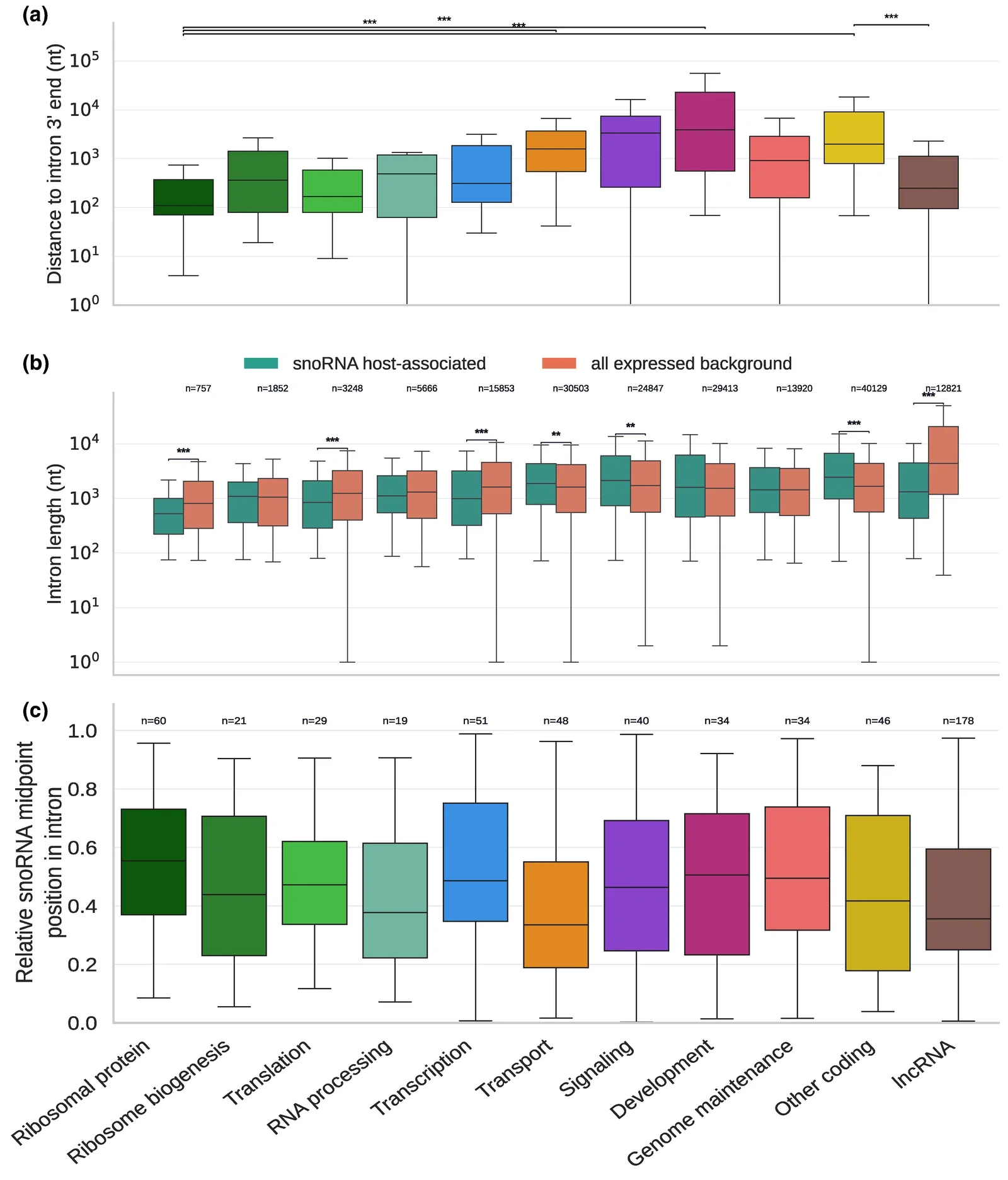
Intron architecture of snoRNAs by host–gene class. Asterisks indicate statistical significance after Benjamini–Hochberg correction: * adjusted *P* < 0.05, ** adjusted *P* < 0.01, and *** adjusted *P* < 0.001. a) Distribution of the absolute distance between the snoRNA 3′ end and host-intron 3′ boundary. b) Lengths of snoRNA-containing introns compared with expressed background introns from the same host classes; the color key in (b) distinguishes snoRNA host-associated introns from expressed background introns. Sample sizes and adjusted *P*-values are shown. c) Transcript-oriented normalized intronic position, calculated from the intron 5′ boundary to the snoRNA midpoint and divided by full intron length, where 0 is the 5′ boundary and 1 the 3′ boundary (total *n* = 560; *n* = 19–178 per class). Box plots show medians and interquartile ranges. No pairwise comparison remained significant after Benjamini–Hochberg correction (minimum adjusted *P* = 0.294).

A similar pattern was observed for host intron length. SnoRNAs in ribosomal protein genes were embedded in much shorter introns (median 522 nt) than snoRNAs in signaling genes (2,130 nt) or development genes (1,602 nt) (Fig. 2b). These differences indicate that ribosome-related host genes provide a compact intronic environment, whereas other host classes are associated with much larger intronic regions. Longer introns may introduce additional regulatory complexity or reduce processing efficiency, potentially contributing to lower abundance levels in these classes (as indeed observed in Fig. 3). Intriguingly, snoRNA-encoding introns of transport, signaling, development, and “other coding” genes are significantly longer than non-snoRNA-encoding introns of these gene classes, suggesting that this group of snoRNAs is under selective pressure to be hosted in this type of environment.

**Fig. 3.**
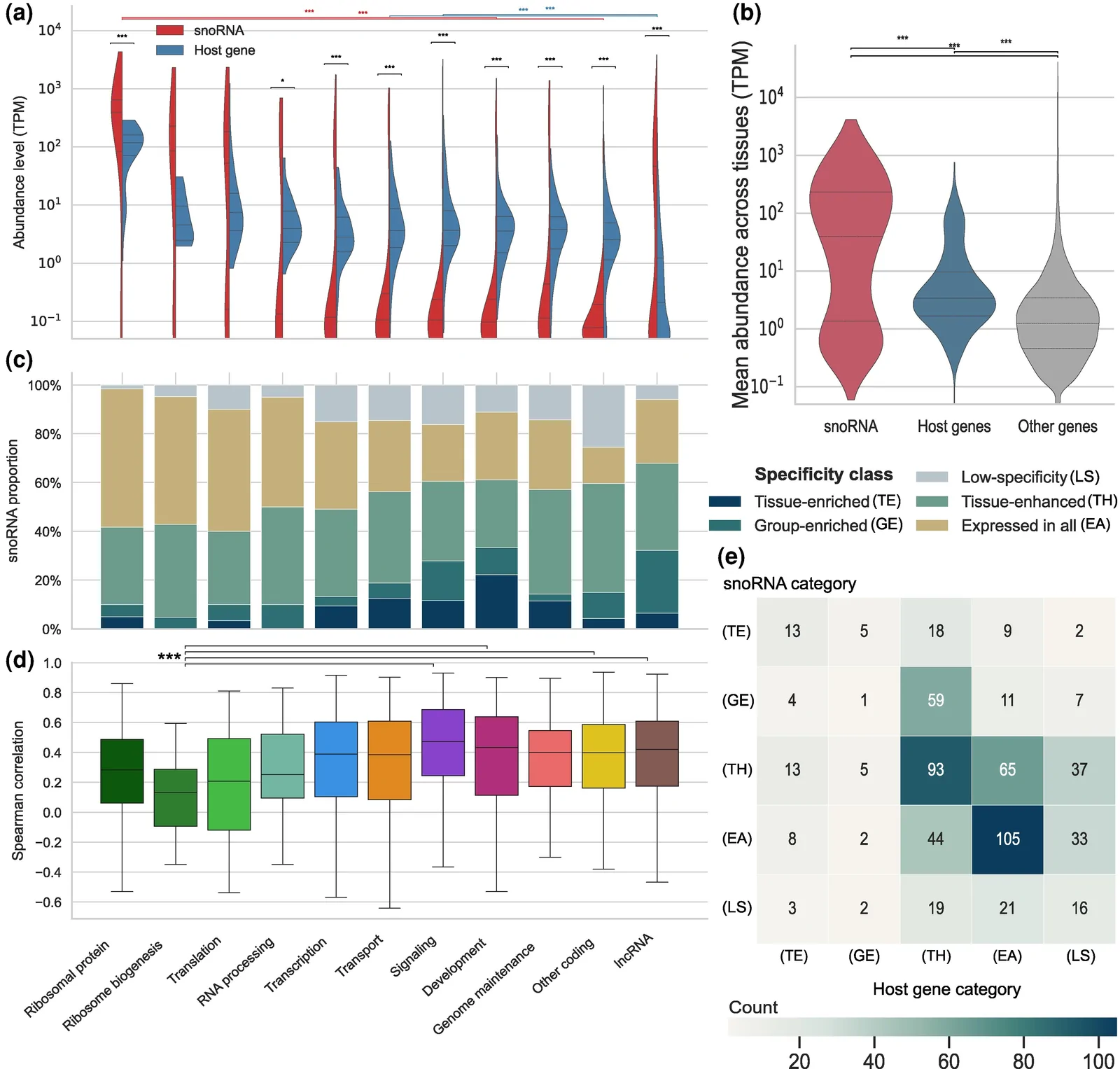
Abundance relationships between human snoRNAs and host transcripts. Asterisks indicate statistical significance after Benjamini–Hochberg correction: * adjusted *P* < 0.05, ** adjusted *P* < 0.01, and *** adjusted *P* < 0.001. a) Distributions of tissue-averaged snoRNA and mature host-transcript abundance by host–gene class. b) Abundance distributions for snoRNAs, their mature host transcripts, and other expressed human genes. c) Tissue-abundance specificity categories by host class. The color legend is to the right of the figure. d) Spearman correlations between snoRNA and host-transcript abundance across tissues. These correlations describe abundance covariation and do not directly measure transcription. e) Counts of snoRNA–host pairs by tissue-abundance specificity category. Sample sizes are displayed on the corresponding plots.

To account for intron length, we compared normalized intronic positions (Fig. 2c). Among 560 snoRNAs fully contained within annotated introns, median fractions ranged from 0.335 in transport hosts and 0.355 in lncRNA hosts to 0.554 in ribosomal protein hosts (*n* = 19–178 per class). No pairwise comparison remained significant after Benjamini–Hochberg correction (minimum adjusted *P* = 0.294).

Taken together, these results indicate that snoRNAs are not only associated with specific host gene types but also with distinct structural environments. Ribosomal protein genes, and to a lesser extent ribosome biogenesis, translation, and lncRNA host genes, combine short introns and close snoRNA positioning relative to splice sites, whereas many other host classes provide more extended genomic contexts. These architectural differences may contribute to variation in snoRNA processing efficiency and expression.

### Abundance Coupling Between snoRNAs and Their Host Genes

Beyond structural constraints imposed by host gene architecture, we next investigated how these genomic contexts translate into their abundance patterns. This analysis aimed to determine to what extent snoRNA abundance reflects host gene transcriptional activity and whether this relationship varies depending on host gene context.

SnoRNA abundance differed strongly across host–gene classes (Fig. 3a; Figs. S2 to S4). SnoRNAs hosted by ribosomal protein genes were the most abundant (median 390.5 TPM), followed by those in ribosome biogenesis (86.4 TPM) and translation genes (52.6 TPM); most other classes had medians below 1 TPM. Host-transcript abundance showed a partly similar ordering, with medians of 118.1 TPM for ribosomal protein genes, 7.49 TPM for translation genes, and 4.60 TPM for ribosome biogenesis genes, whereas the remaining coding classes ranged from 2.54 to 3.96 TPM. LncRNA hosts spanned a broad range (0–3,861 TPM). These distributions show an association between host class and host gene RNA accumulation. At the host-gene level, host gene transcripts also showed higher abundance than transcripts from other human genes that do not host snoRNAs (median 3.39 vs. 1.25 TPM; Fig. 3b). This suggests that snoRNAs are preferentially embedded within transcriptionally active loci, rather than being randomly distributed across the genome. However, this analysis does not distinguish higher transcription from increased transcript stability or other posttranscriptional effects.

Tissue-specificity analysis further highlighted these differences. To conduct this analysis, we applied the human protein atlas tissue specificity definitions (Uhlén et al. 2015) and considered snoRNA and host gene abundance across 11 normal human tissues. SnoRNAs embedded within ribosomal protein, ribosome biogenesis, and translation genes were predominantly associated with broadly expressed categories, whereas snoRNAs in signaling, development, other coding, and lncRNA host genes were more frequently classified as tissue-enhanced, tissue-enriched, or group-enriched (Fig. 3c). Thus, ribosome and translation-related host genes are linked to constitutive snoRNA expression across tissues, reflecting their housekeeping functions and higher conservation (Fig. S5), while other host gene classes are associated with more specialized abundance patterns.

The same broad ordering was observed across individual tissues: snoRNAs in ribosomal protein and ribosome biogenesis genes were generally among the most abundant, whereas lncRNA-hosted and other nonribosomal snoRNAs were more variable (Figs. S2 and S3). The pattern is therefore not attributable to a single tissue in this dataset.

To assess abundance covariation, we calculated Spearman correlations between snoRNA and host–transcript abundance across tissues (Fig. 3d). Median correlations were positive but modest and varied from 0.13 in ribosome biogenesis genes to 0.47 in signaling genes. Even when both RNA products were abundant, as in ribosomal protein hosts, correlations were moderate rather than strong. The median correlation was lower for ribosome biogenesis than for ribosomal protein hosts (*P* = 0.019), as previously reported (Fafard-Couture et al. 2021). These correlations do not directly measure transcriptional coupling.

The variation in correlation strength indicates that mature snoRNA abundance is not reliably predicted by mature host–transcript abundance. It is consistent with heterogeneous relationships among RNA synthesis, processing, and stability across host contexts.

Tissue-abundance categories were likewise discordant for many snoRNA–host pairs (Fig. 3e). Broadly detected snoRNAs could be paired with tissue-enhanced or tissue-enriched host transcripts, and several other cross-category combinations occurred. Differences in splicing efficiency, intron processing, snoRNA stability, host–transcript stability, and nonsense-mediated decay could contribute to these mismatches.

Taken together, these results show heterogeneous abundance relationships between snoRNAs and the transcripts of their hosts. Intronic biogenesis requires production and processing of the host precursor RNA, but the measured abundances can additionally reflect differential processing, stability, and decay. Direct conclusions about transcription would require nascent-transcription measurements.

### Host Gene Diversity Within snoRNA Families

We next investigated whether genomic context was associated with snoRNA multiplicity and host-class retention within expressed snoRNA families. We considered all Rfam snoRNA families with at least one expressed human member, for a total of 217 families covering 538 expressed human snoRNAs (81.3% of all expressed human snoRNAs). Of these, 128 consisted of only one expressed member in human and were concentrated in lncRNA, ribosomal protein, and translation hosts (41, 19, and 16 families, respectively; Fig. S6a). For the 89 families with more than one expressed member in human, host-context retention varied with the dominant host gene class. Families dominated by RNA processing, lncRNA, or ribosomal protein hosts showed the strongest retention, remaining restricted to a single host class in 100%, 60.0%, and 47.6% of cases, respectively, with median dominant-host shares of 1.00, 1.00, and 0.75, respectively (Fig. 4 and Fig. S6b). In contrast, signaling- and transcription-dominated families were usually dispersed across multiple host classes (restricted in 14.3% and 27.3% of cases). Overall, 41.6% of multimember families were confined to one host gene class. This class-level coherence was also snoRNA-type dependent: 60.9% of multimembered C/D families, but only 20.5% of multimembered H/ACA families, were restricted to one host gene class. This is coherent with our previous findings (Bergeron et al. 2021) but should however be interpreted cautiously with the small sample sizes as shown in Fig. S6.

**Fig. 4.**
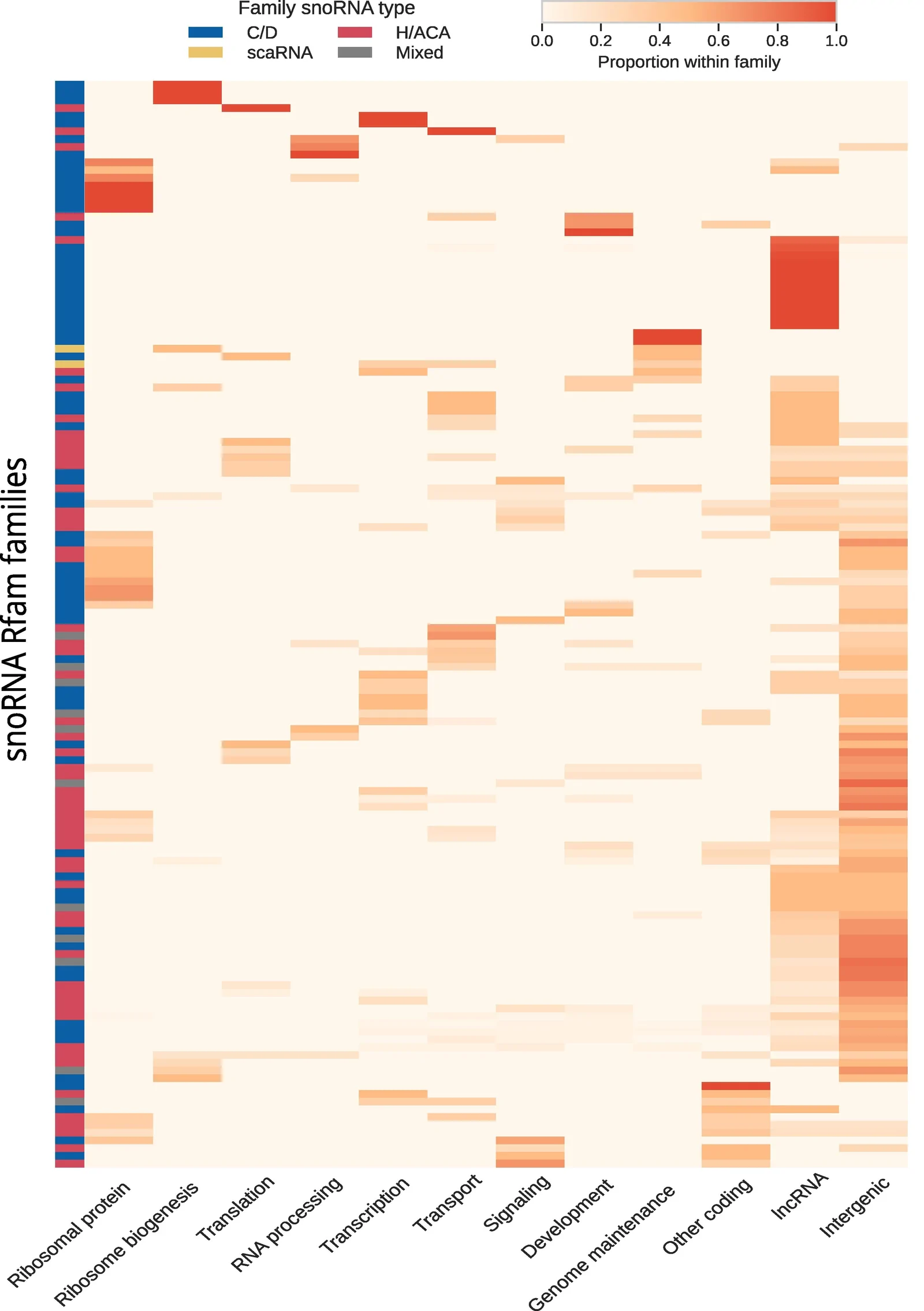
Heterogeneity of host-gene classes within snoRNA families. Heatmap showing, for each Rfam family with at least 2 human members and at least one expressed member, the fraction of expressed members assigned to each host–gene class. Rows are families and columns are operational host classes; color intensity gives the within-family fraction. Family and class sample sizes are shown in Fig. S6, which omits intergenic display categories and shows only host classes with at least 2 multimember families in (b); categories represented by few multimember families should be interpreted cautiously.

Together, these results indicate that snoRNA family composition and host–class retention are associated with host context. Consequently, understanding how these class-specific genomic environments are preserved or reshaped across species becomes essential to interpret the evolutionary conservation of snoRNA–host gene relationships.

### Evolutionary Conservation of snoRNAs and Host–Gene Relationships

If snoRNA function and host context are linked, host–gene or host–class associations may be retained across species. We therefore retrieved Ensembl orthologs of human snoRNAs and assigned each ortholog to its annotated host locus. When several candidates were available for a snoRNA–species pair, an exact match to the human host gene was prioritized; among otherwise equivalent candidates, the highest Ensembl percentage-identity score was retained. We compared conservation at 2 levels: retention of the snoRNA sequence and stability of its genomic context.

At the sequence level, expressed human snoRNAs were broadly conserved, with a median of 55 species containing an equivalent ortholog snoRNA out of 197 species considered (Fig. S7). This widespread conservation reflects the fundamental roles of many snoRNAs in RNA modification, particularly in rRNA and snRNA processing. Because these functions are essential and highly conserved across eukaryotes, strong evolutionary constraint on snoRNA sequences is expected.

In contrast to the broad conservation of snoRNA presence across vertebrates, conservation of the *snoRNA–host gene pairing* was markedly limited. Figure 5a shows that many human snoRNAs have orthologs in numerous species, indicating that the presence of the snoRNA itself is frequently maintained across evolution. However, Fig. 5a and b reveal that these orthologous snoRNAs are often not associated with the same host gene across species. In most cases, orthologs are found in different genes or even different host gene types, indicating that conservation of the *exact genomic embedding* is uncommon.

**Fig. 5.**
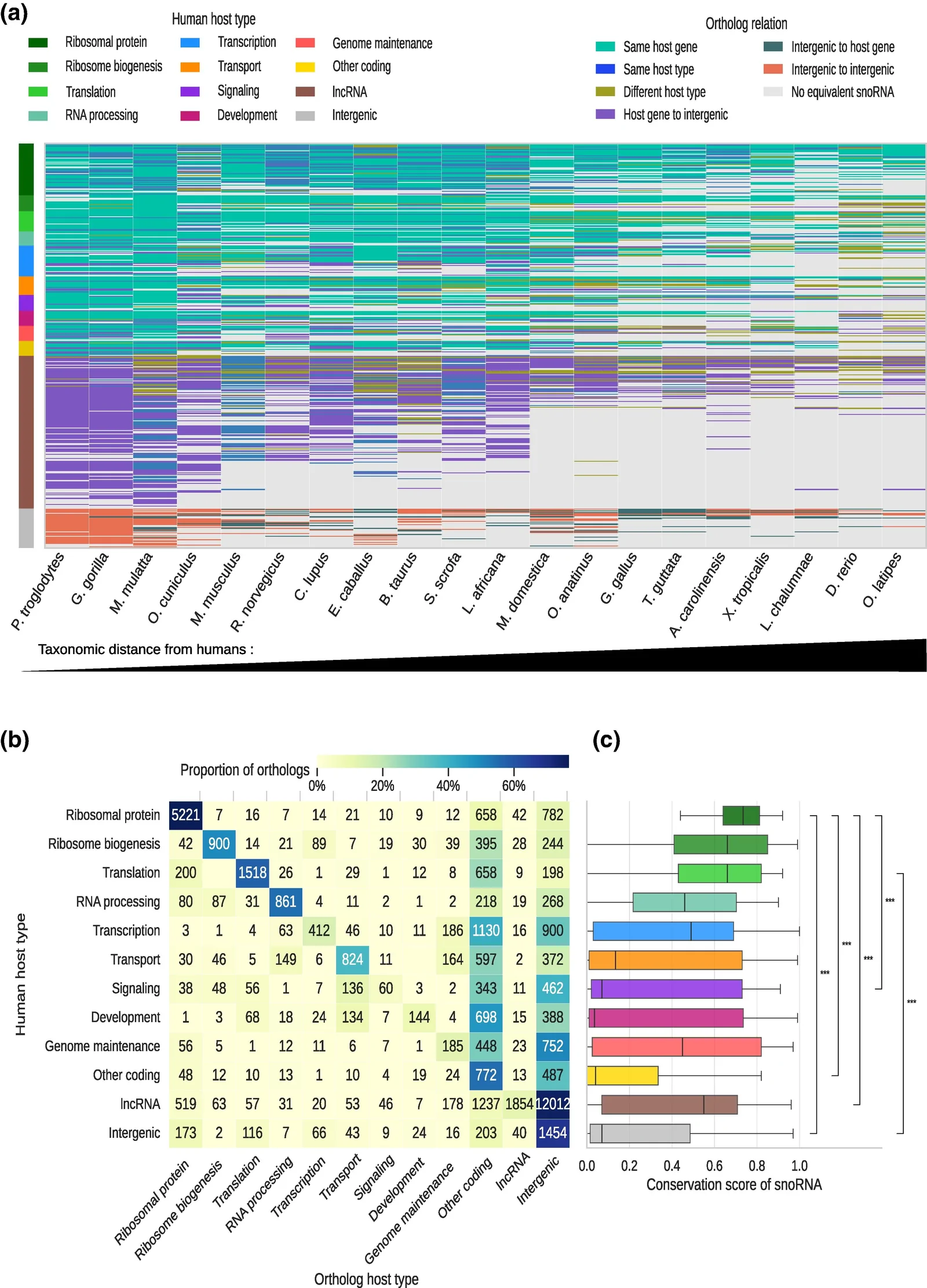
SnoRNA sequence conservation and host-context stability across vertebrates. a) Heatmap for 459 expressed human snoRNAs with at least one retained ortholog across 20 well-annotated vertebrates: chimpanzee, gorilla, macaque, rabbit, mouse, rat, dog, horse, cattle, pig, African elephant, opossum, platypus, chicken, zebra finch, anole, Xenopus, coelacanth, zebrafish, and medaka. Colors distinguish 7 ortholog–host relationships; gray denotes no retained equivalent. b) Quantitative summary across all retained species of same-host-gene, same-host-class, different-host-class, and intergenic relationships, demonstrating that snoRNA sequence retention and exact host–gene retention are distinct. Sample sizes are shown by human host class. c) Distribution of mean UCSC 100-vertebrate PhastCons scores by host class; medians and adjusted *P*-values are shown in the corresponding plot or supplementary tables (* adjusted *P* < 0.05, ** adjusted *P* < 0.01, and *** adjusted *P* < 0.001).

Accordingly, a low proportion of orthologs in the same host gene does not necessarily indicate loss of the snoRNA. It instead records host-context turnover among the annotated orthologs, with substantial variation among snoRNAs and host classes.

The observed patterns are consistent with partly distinct constraints on snoRNA sequence or target recognition and on genomic embedding. Host-context stability nevertheless differed among classes. For snoRNAs hosted by human ribosomal protein genes, 77% of annotated orthologs remained in a ribosomal protein host context (Fig. 5b), although many changed the exact ribosomal protein host. Thus, canonical snoRNAs were frequently retained within a functionally similar host class even when exact host identity differed. This association is compatible with selection on snoRNA function and host environment.

SnoRNA sequence conservation also varied by human host context. Median 100-vertebrate PhastCons scores were 0.735 for ribosomal protein hosts and 0.660 for both ribosome biogenesis and translation hosts, compared with lower values for intergenic snoRNAs and several nonribosomal classes (Fig. 5c). These differences associate ribosome-related host contexts with stronger sequence conservation but may partly reflect conservation of the surrounding host loci.

To examine potential confounding by host–locus conservation, we compared up to 500 nt of the exons flanking each snoRNA-containing intron. SnoRNA–host gene associations across vertebrates were classified as conserved when more than 50% of all available orthologs retained the human host gene, and nonconserved otherwise. Flanking exons were more conserved overall for conserved than for nonconserved snoRNA–host gene associations (*n* = 115 and 288; median PhastCons = 0.928 and 0.101; Mann–Whitney *U P* = 1.62 × 10^−25^). However, none of the ten within-class comparisons remained significant after Benjamini–Hochberg correction (Fig. S8). Thus, host–locus conservation covaries with pairing stability in the pooled comparison; this comparison is not a multivariable adjustment and cannot establish an independent effect of snoRNA role or host class.

Together, these results show that snoRNA sequence conservation generally exceeds conservation of exact host–gene identity and that both measures vary among operational host classes. The observational analyses do not isolate an independent causal effect of host context and do not distinguish the evolutionary mechanisms that generated them.

### Case Studies of Conserved and Dynamic snoRNA–Host Gene Relationships

To illustrate trajectories represented in the global analysis, we examined selected snoRNA loci and families. These cases were chosen as examples of stable, intermediate, and dynamic host associations and do not constitute evidence independent of the dataset-wide comparisons.

Some snoRNAs retained the same host gene across many vertebrates. Within EIF4A2, for example, SNORD2 had the same annotated host gene in 84 of 109 species containing an ortholog (Fig. 6a). This pattern is concordant with the cis-regulatory interaction previously described between SNORD2 and EIF4A2 (Bergeron et al. 2023a) while remaining an illustrative single-locus example.

**Fig. 6.**
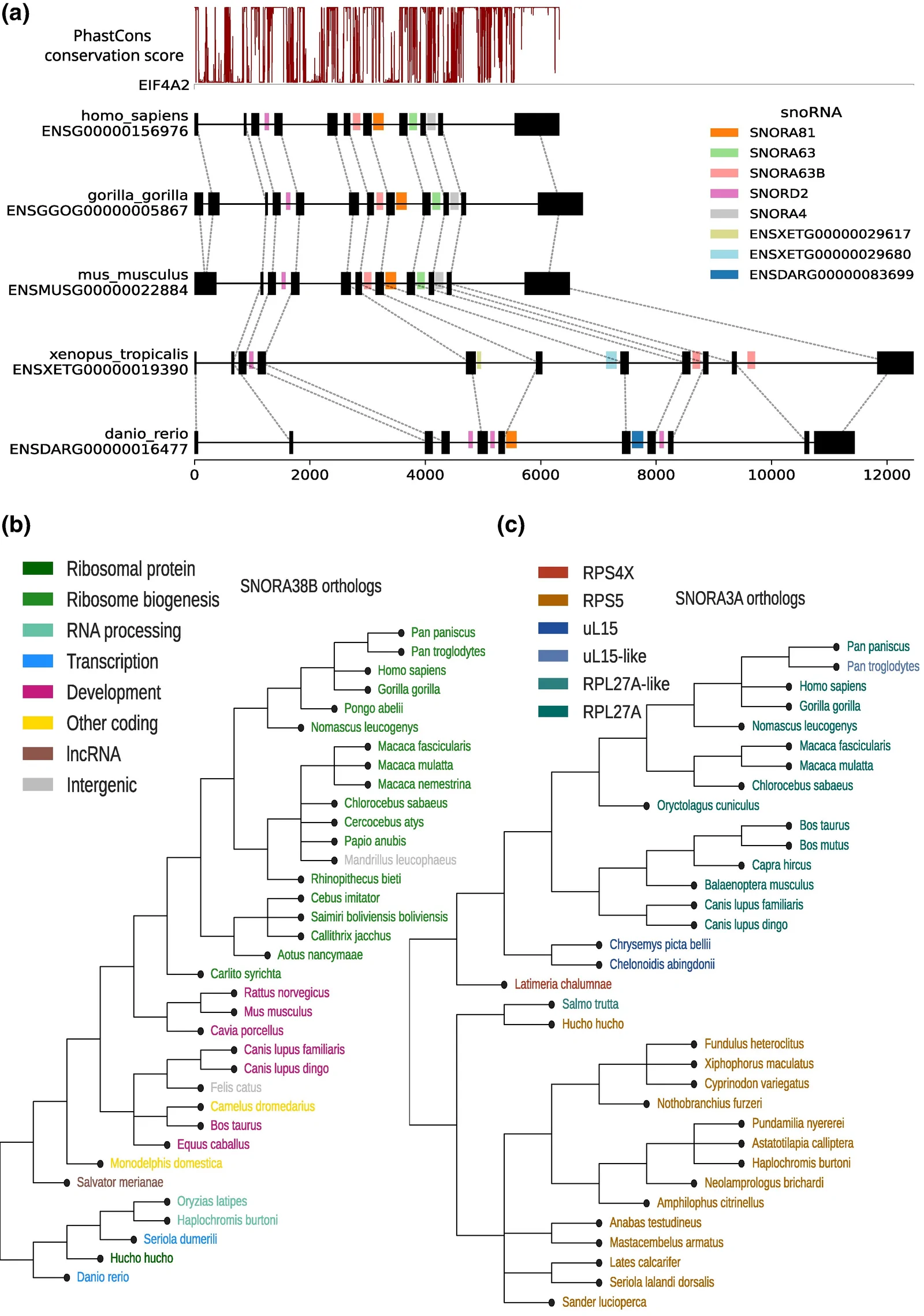
Illustrative snoRNA–host–gene relationships across vertebrates. a) Scaled genomic view of the EIF4A2 locus across selected species, with position 0 at the first exon and the endpoint at the last exon. Ensembl-canonical transcripts were used when available, and conservation is shown for the human gene with the 100-vertebrate PhastCons track. (b, c) Phylogenetic trees for SNORA38B b) and SNORA3A c) orthologs in selected vertebrates. Label colors indicate host–gene class b) or specific ribosomal protein host (c). When several candidates were available for a snoRNA–species pair, an exact match to the human host gene was prioritized; among otherwise equivalent candidates, the highest Ensembl perc_id was retained. These panels illustrate trajectories selected from the global analysis and are not independent tests.

SNORA38B illustrates greater host-context turnover, with orthologs assigned to 8 host–gene classes (Fig. 6b). The analysis establishes changes in annotated host association but does not identify the underlying mechanism. SNORA38B has canonical rRNA and U4 snRNA targets and has also been implicated in an E2F1-dependent GAB2/AKT/mTOR pathway in lung cancer (Zhuo et al. 2022), illustrating that a snoRNA's reported functions need not mirror those of every host context.

SNORA3A provides an intermediate example: 86.9% of ortholog-containing species retained a ribosomal protein host context although not always the same host gene (Fig. 6c). In humans, this canonical H/ACA guide is embedded in RPL27A and is associated with several rRNA pseudouridylation sites. Its host-class retention is consistent with functional concordance but does not by itself demonstrate that the host class is required for snoRNA function.

These selected cases illustrate the range of host-association patterns summarized by the global analyses; they should not be interpreted as an independent test of the proposed conceptual continuum.

### A Gradient of snoRNA–Host–Gene Relationships

For a descriptive synthesis, we defined 3 operational groups of expressed, hosted snoRNAs and compared them with an other background group (Fig. 7a). The bidirectional group comprised snoRNAs previously reported to interact with and regulate their own host transcript while also being functionally coupled with their host gene (as defined for the Functional-coupling group, next sentence), and they represent the snoRNA–host gene pairs with strongest coupling. After excluding those snoRNAs, the functional-coupling group represents snoRNAs that function in the same biological process as the protein encoded by their host gene. This group consists of either an rRNA-targeting snoRNA in a ribosomal protein, ribosome biogenesis, or translation host, or an snRNA-targeting snoRNA in an RNA-processing host, although we note that as more functions are discovered for snoRNAs, more functional couplings can be uncovered for pairs of snoRNAs and host genes. The remaining expressed, hosted snoRNAs form the Passenger group: they presumably use their host gene to ensure their expression but are not otherwise connected with their host gene. The Other group contains intergenic and undetected snoRNAs. Conservation levels across vertebrates of snoRNAs belonging to the 2 most strongly connected snoRNA–host gene pair groups were significantly higher than those of the other groups (median PhastCons scores were 0.72 and 0.72 for the Bidirectional and Functional coupling groups, respectively, but only 0.35 and 0.03 for the Passenger and Other groups). All comparisons involving other or passenger groups were significant after Benjamini–Hochberg correction (adjusted *P* = 6.24 × 10^−39^ to 1.18 × 10^−4^), whereas functional coupling and bidirectional relationships did not differ (adjusted *P* = 0.347). Because the definitions incorporate target and host annotations, these comparisons describe the selected groups rather than independently validating a biological classification.

**Fig. 7.**
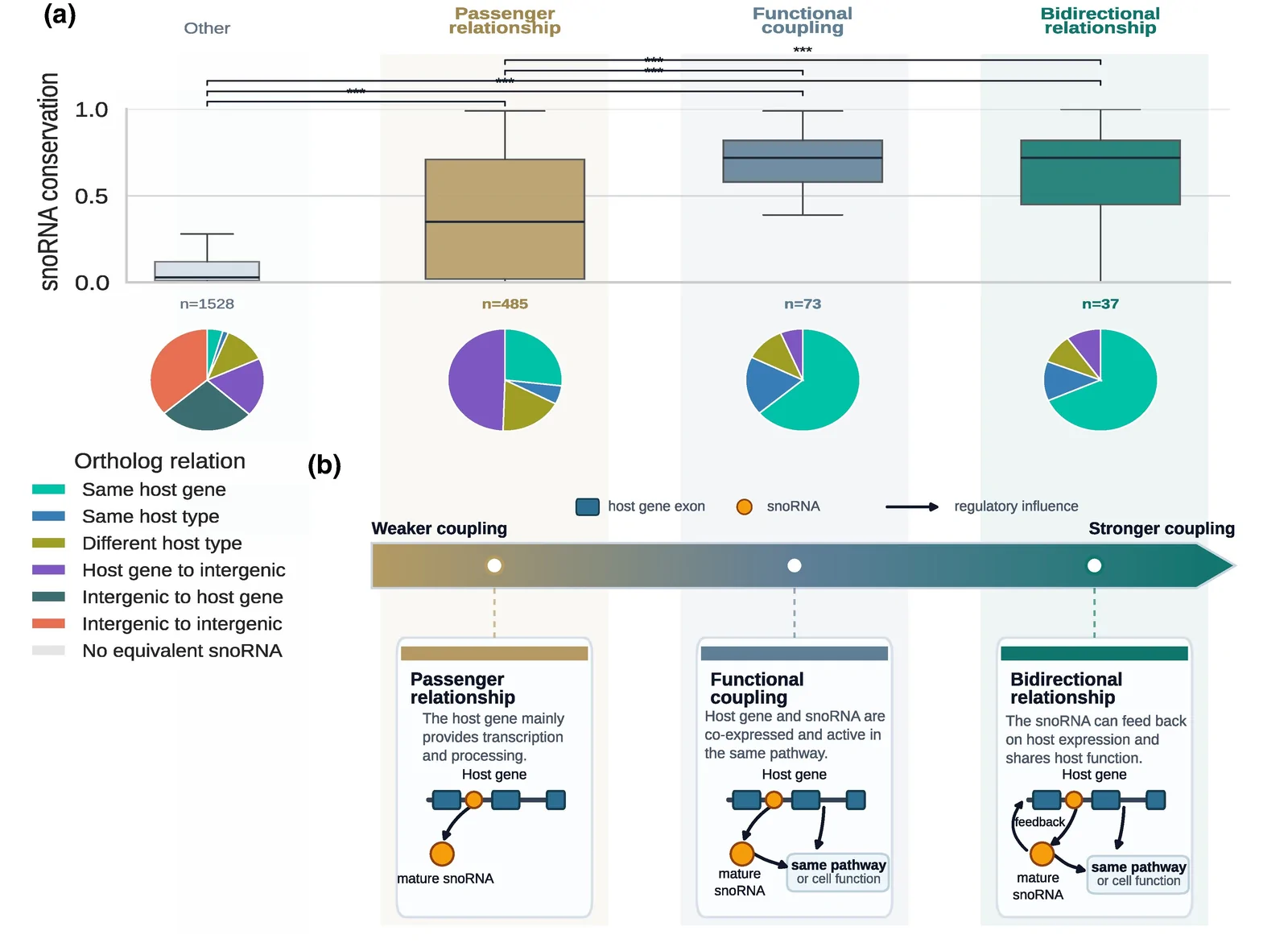
Conceptual synthesis of snoRNA–host-gene relationships. a) Four operational groups were compared: the bidirectional relationship group consisting of snoRNAs reported to interact with their host transcript while also sharing a functional role together (*n* = 37); the Functional coupling group, consisting of snoRNAs sharing a functional role with their host gene (*n* = 73); the Passenger group, consisting of expressed snoRNAs with no regulatory or functional relationship with their host gene (*n* = 485); and the Other group, for intergenic and undetected snoRNAs (*n* = 1,528). Box plots show distributions of mean 100-vertebrate PhastCons scores; pie charts show ortholog–host relationships. All pairwise comparisons were significant after Benjamini–Hochberg correction except functional coupling versus bidirectional relationships (adjusted *P* = 0.347) (* adjusted *P* < 0.05, ** adjusted *P* < 0.01, and *** adjusted *P* < 0.001). b) Conceptual range from passenger arrangements through functional coupling to bidirectional regulation. It does not assert discrete natural classes, causality, or a mechanism of host switching.

Ortholog-context profiles also differed among these operational groups (Fig. 7a). Same-host-gene relationships represented, respectively, 4.45%, 26.84%, 63.74%, and 68.39% of annotated ortholog relationships in Other, Passenger, Functional coupling, and Bidirectional groups, respectively; including same-host-class relationships increased these values to 6.07%, 32.70%, 82.33%, and 80.90%. Host-to-intergenic relationships represented 18.61%, 49.46%, 6.36%, and 9.83%. Thus, snoRNAs involved in regulating the maturation of their host gene (Bidirectional group) or playing roles in the same biological processes as their host gene (Functional coupling group) are more likely to be conserved across vertebrates and to have a stable relationship with their host gene, or at least to remain evolutionarily associated with host genes of the same type than all other snoRNAs. These associations motivate the conceptual model in Fig. 7b, but they do not establish a causal sequence or discrete natural classes.

## Discussion

SnoRNAs are traditionally studied through the lens of their molecular function in ribosome biogenesis and RNA modification. However, because most vertebrate snoRNAs are encoded within introns of host genes, their genomic organization inherently links snoRNA production to host gene transcription and splicing. In this study, to systematically investigate the relationships between snoRNAs and their host genes, we categorized human snoRNA host genes based on their functions and then compared snoRNA genomic organization, abundance, and conservation across these classes. Because gene functions can overlap and the classification requires tie rules, the 11 classes we propose are useful analytical summaries rather than mutually exclusive biological entities. Our analyses show that the genomic relationship between snoRNAs and host genes is associated with distinct patterns of snoRNA distribution across host gene classes. In particular, ribosomal protein genes and regulators of ribosome biogenesis are strongly enriched for snoRNAs compared with the genome-wide gene distribution, and these genes frequently contain multiple snoRNAs (Fig. 1). This organization is consistent with the hypothesis that certain host genes provide specialized genomic environments favoring the accumulation and coordinated expression of multiple snoRNAs involved in ribosome-related functions.

The architecture of introns hosting snoRNAs also differs among host gene classes. SnoRNAs embedded in ribosomal protein genes and lncRNA host genes tend to lie at shorter absolute distances from the 3′ end of their introns and within shorter introns compared with many other gene types (Fig. 2). Because efficient maturation of box C/D snoRNAs depends on their positioning relative to spliceosomal elements such as the branch point (Hirose and Steitz 2001; Yang 2015), these structural features may create genomic contexts that favor efficient snoRNA processing. Such architectural constraints may partly explain the higher abundance of snoRNAs in ribosomal protein hosts and suggest that intron structure contributes to shaping the genomic landscape of snoRNA distribution. Nevertheless, our data do not measure processing kinetics, and differences in intron length can affect absolute-distance comparisons. The normalized position measure addresses that geometric issue but does not establish that intron architecture causes differences in mature snoRNA abundance.

Abundance analyses further reveal that snoRNA–host–gene relationships are heterogeneous. SnoRNAs located within ribosomal protein and ribosome biogenesis genes generally display high abundance levels and broad tissue distribution, consistent with the housekeeping nature of ribosome production. In contrast, snoRNAs embedded in other host gene classes show more variable expression profiles. Correlations with mature host-transcript abundance are modest and differ among classes, without uniformly stronger correlations in ribosome-related hosts (Fig. 3). These observations are consistent with previous transcriptome-wide analyses showing that snoRNA abundance is often only partially coupled to host gene expression and can be uncoupled by posttranscriptional mechanisms such as alternative promoter usage, nonsense-mediated decay, and differential intron processing (Lykke-Andersen et al. 2014; Fafard-Couture et al. 2021; Fafard-Couture et al. 2024). Our results extend those studies by showing that the degree of coupling is itself structured by host gene class.

Beyond these abundance relationships, the greater conservation of canonical snoRNAs in ribosome-related contexts is consistent with the hypothesis that noncoding RNAs acting in ancient, broadly conserved processes may experience stronger evolutionary constraint than RNAs involved in more lineage-specific functions (Metzinger and Metzinger-Le Meuth 2026). As proposed for lncRNAs, functional conservation need not require retention of the complete primary sequence or exact genomic position; for snoRNAs, preservation of target recognition and a compatible host–gene class may be more consequential than preservation of the exact host gene. Our comparison of adjacent host exons showed that conserved snoRNA–host gene associations occur in more conserved loci (Fig. S8), indicating that host–locus conservation covaries with pairing stability.

We found snoRNA—host gene associations across vertebrates to be heterogeneous, spanning highly stable relationships all the way to much more recently evolved relationships (Fig. 5). The EIF4A2/SNORD2, SNORA3A, and SNORA38B cases illustrate representative examples of stable, intermediate, and dynamic host associations, respectively (Fig. 6). Indeed, SNORD2 is highly conserved across vertebrates and displays a remarkably stable association with its host gene EIF4A2 (84/109). In contrast, SNORA38B is encoded in a wide variety of genomic contexts across vertebrates, including being housed by genes belonging to 6 different coding host gene classes, by a lncRNA and can also be intergenic across vertebrates. SNORA3A is an intermediate but still intriguing example, always encoded in ribosomal protein host genes across vertebrates but housed in diverse ribosomal protein genes both of the small and large subunits of the ribosome. These case studies were selected from the same global dataset and therefore serve as examples rather than independent validation. It is important to note that observed host turnover does not identify the evolutionary mechanism that produced a change in genomic embedding.

Together, the global analyses and these illustrative examples indicate that snoRNA–host gene relationships cannot be described as a single uniform mode of interaction. Instead, our results suggest the existence of a spectrum of relationships between snoRNAs and their host genes, reflecting varying degrees of regulatory and functional coupling (Fig. 7). At one end of this spectrum, snoRNAs embedded within ribosome-related genes appear to form tightly integrated genomic units in which host gene transcription, intronic architecture, and snoRNA function may be coordinated. In these cases, the host gene environment may provide favorable conditions for snoRNA expression and for fulfilling essential roles in ribosome biogenesis. In the most strongly coupled pairs, not only do snoRNAs and the product of their host genes function in the same biological process, but they can also regulate each other at the level of their biogenesis, as has been shown for SNORD86 and SNORD2 (Lykke-Andersen et al. 2018; Bergeron et al. 2023a).

Intermediate situations also exist, in which snoRNAs and host genes function in the same biological pathways but display weaker coupling of RNA abundance. In such cases, snoRNAs may participate in broader RNA regulatory networks while residing within genes involved in related pathways, such as ribosomal protein production or ribosome biogenesis. Finally, some snoRNAs show little functional or regulatory association with their host genes. These loci may represent genomic contexts in which host genes primarily act as transcriptional carriers enabling snoRNA production rather than forming coordinated functional units.

Based on these observations, we propose a gradient model of snoRNA–host gene relationships (Fig. 7b). In this model, snoRNAs occupy positions along a continuum ranging from strongly coupled systems—where snoRNA expression and function are tightly integrated with host gene biology—to weakly associated passenger snoRNAs that show minimal functional or regulatory association with their host genes. Movement along this continuum may occur through evolutionary processes such as gene duplication, recombination, or retrotransposition that relocate snoRNAs into new genomic environments, although the observed host-association changes do not distinguish among these mechanisms.

This gradient should be read as a conceptual synthesis that provides a vocabulary for passenger arrangements, functional coupling, and bidirectional regulation. Its operational groups incorporate prior information about snoRNA targets, host functions, and reported host-transcript interactions. The associated conservation differences are descriptive of those definitions and do not show that the categories emerged unsupervised from the data. Intronic snoRNAs require production and processing of a host precursor RNA, but mature snoRNA and host-transcript abundances can diverge because their processing and decay differ.

Overall, snoRNA sequence conservation, host-context stability, intron architecture, and abundance are associated but nonequivalent properties. Future analyses using nascent-transcription data and experimental measurements of processing and stability will be required to distinguish causal contributions from shared functional or evolutionary constraints.

## Materials and Methods

### Data Acquisition and Annotation Sources

All analyses were performed using the human genome assembly GRCh38 (hg38). Human snoRNA annotations were obtained from snoDB v2.0 (Bergeron et al. 2023b). Reference gene annotations for all species were retrieved from Ensembl release 114 in GTF format (Dyer et al. 2025). Host gene annotations and genomic features were extracted from these files for downstream analyses. Gene metadata and Gene Ontology (GO) annotations used for host gene classification were retrieved from MyGene.info v3 (Xin et al. 2016; fields: symbol, name, taxid, type_of_gene, ensembl, go.BP, go.MF, and go.CC; accessed on January 8, 2025). GO ancestry relationships were evaluated using the Gene Ontology go-basic.obo release downloaded from current.geneontology.org.

### Host Gene Assignment

Host genes were assigned to snoRNAs by strand-specific genomic overlap with Ensembl gene annotations using bedtools intersect (v2.31; Quinlan and Hall 2010). Partial overlaps were considered sufficient for assignment in orthologous genomes but not in the human reference.

Intronic regions for Fig. 2 were defined from the Ensembl-canonical transcript when available. Introns corresponded to the genomic intervals between annotated exons. When no canonical transcript was available, the shortest annotated transcript was used to define exon–intron structure.

SnoRNAs not overlapping any annotated gene were classified as intergenic.

### Host Gene Classification

Host genes were classified with a custom Python workflow (https://github.com/scottgroup/Classification_Genes) based on normalized Ensembl identifiers, MyGene.info annotations, and GO graph traversal. Ribosomal protein genes were identified first by ancestry to structural constituent of ribosome (GO:0003735) or strict symbol and long-name patterns for structural ribosomal proteins. Genes annotated as lncRNA or lincRNA in the Ensembl-linked type_of_gene field were assigned directly to the lncRNA class.

For host genes not belonging to the ribosomal protein gene or lncRNA classes, GO terms were propagated upward through is_a, part_of, regulates, positively_regulates, and negatively_regulates relationships. Biological process anchors defined ribosome biogenesis (GO:0042254), translation (GO:0006412), RNA processing (GO:0006396), transcription (GO:0006351 and GO:0001172), transport (GO:0006810), development (GO:0032502), signaling (GO:0023052 and GO:0007154), and genome maintenance (GO:0007049 and GO:0006259). Molecular function anchors additionally supported transcription (GO:0140110) and genome maintenance (GO:0003678, GO:0003684, GO:0003697, GO:0003887, GO:0017108, and GO:0140658). Each gene was assigned to the class with the greatest number of biological process and molecular function annotations whose ancestry reached at least one class anchor. When no informative GO anchor was recovered, curated gene-symbol patterns provided a conservative fallback for selected classes; remaining protein-coding hosts were assigned to other coding. Ties followed the predefined priority order: ribosomal protein, ribosome biogenesis, translation, RNA processing, transcription, genome maintenance, transport, development, signaling, and other coding.

### Host-gene Classification Diagnostics

For host genes assigned to the 8 GO-based functional classes, excluding ribosomal protein genes, classification support was summarized as the number and fraction of informative biological process GO annotations reaching each class anchor. These fractions were nonexclusive because a GO annotation could reach more than one anchor. In the diagnostic table, 218 host genes had classifiable biological process support; the median assigned-class support fraction was 0.95 (interquartile range 0.55–1.00). Overall, 81.7% of host genes were unambiguously attributed to their classes (Fig. S1).

These diagnostic summaries were used to identify host classes for which annotation ambiguity or small sample size requires cautious interpretation. They are reported in Fig. S1 and the Supplementary material together with class-specific sample sizes.

### SnoRNA Target Biotype Identification

Only biologically validated snoRNA targets curated in snoDB (Bergeron et al. 2023b) were used. These annotations were derived from snoRNABase and from experimentally supported snoRNA-target interactions reported in the literature (Lestrade and Weber 2006; Kehr et al. 2014; Krogh et al. 2016; Vitali and Kiss 2019; Zhang et al. 2023; Liu et al. 2025; Song et al. 2025).

### RNA Sequencing Data and Expression Quantification

RNA sequencing data were obtained from previous TGIRT-seq studies, a low-structure-bias approach employing thermostable group II intron reverse transcriptases that provide accurate representation of highly structured and modified RNAs such as snoRNAs (Boivin et al. 2018). All libraries were generated from total ribodepleted RNA without poly(A) selection. SnoRNA and host-transcript abundances were quantified from the same TGIRT-seq libraries. Sequencing files were retrieved from the Gene Expression Omnibus or Sequence Read Archive under accessions GSE126797, GSE157846, and PRJNA1478292.

Reads were processed with a pipeline designed to quantify embedded and multi-copy genes (https://github.com/scottgroup/snorna_exprof.git). Quality control used FastQC v0.11.9 before and after trimming, fastp (Chen et al. 2018) for trimming, and MultiQC (Ewels et al. 2016) for aggregation. Reads were aligned to GRCh38 with STAR v2.7.9a (Dobin et al. 2013) using --runMode alignReads, --readFilesCommand zcat, --outFilterType BySJout, --outStd Log, --outSAMunmapped None, --outSAMtype BAM SortedByCoordinate, --outFilterScoreMinOverLread 0.3, --outFilterMatchNminOverLread 0.3, --outFilterMultimapNmax 100, --winAnchorMultimapNmax 100, and --alignEndsProtrude 5 ConcordantPair; the STAR index used --sjdbOverhang 74. Reads were assigned with CoCo (Deschamps-Francoeur et al. 2019) to account for nested genes and multimapping, using “coco ca” to generate a corrected GRCh38 annotation and “coco cc --strand 1 --thread 32 --paired” for counting.

Abundances were normalized as transcripts per million (TPM). A gene or snoRNA was considered expressed when its abundance exceeded 1 TPM in at least one tissue. This threshold was retained for consistency with previous snoRNA abundance analyses (Boivin et al. 2018; Fafard-Couture et al. 2021), and sensitivity checks with alternative cutoffs did not change the qualitative conclusions.

### Orthology Inference

Orthologous relationships were retrieved from the Ensembl REST API using the endpoint:

https://rest.ensembl.org/homology/id/human/{ensembl_id}

API queries used requests v2.32.5, and results were parsed with pandas json_normalize. One-to-one and one-to-many relationships were retained. When several candidates were identified for a snoRNA–species pair, an exact match to the human host gene was prioritized; among otherwise equivalent candidates, the ortholog with the highest Ensembl percentage-identity score (perc_id) was used downstream. For Fig. S8, all distinct orthologs were retained.

### Sequence Conservation Analysis

SnoRNA sequence-conservation scores were obtained from the UCSC 100-vertebrate PhastCons hg38 alignment track (Siepel et al. 2005) in bigWig format. For each snoRNA, conservation was calculated as the mean PhastCons score across all nucleotides in its genomic interval.

For the host-locus conservation comparison, up to 500 nt of each exon flanking a snoRNA-containing intron were extracted on both sides (upstream and downstream of the snoRNA), and mean PhastCons coverage was calculated. SnoRNA–host pairings in Fig. S8 with at least 5 informative orthologs were designated conserved when the fraction same_host/(same_host + different_host) exceeded 0.5.

### Genomic Feature Analysis

Distances were calculated from genomic coordinates in transcript orientation. The absolute 3′ distance was measured from the snoRNA 3′ end to the start of the downstream exon. The normalized intronic-position measure was calculated as the distance from the intron 5′ boundary to the snoRNA midpoint divided by full intron length. Thus, 0 represents the intron 5′ boundary and 1 the 3′ boundary. Only snoRNAs fully contained within an annotated intron were retained; 27 partial or noncontained annotations were excluded, leaving 560 snoRNAs.

Intron lengths were derived from the exon–intron structures described above. Comparisons used snoRNA-containing introns and expressed background introns from the corresponding host–gene class; group sizes are reported in Fig. 2 and the Supplementary material.

### Abundance Correlation and Tissue Specificity

Covariation between snoRNA and host-transcript abundance across tissues was assessed with Spearman rank correlation on mean abundance across biological replicates of each tissue, using scipy.stats.spearmanr.

Tissue-specificity categories (for example, tissue-enriched and broadly detected) were defined from tissue-level TPM distributions following the Human Protein Atlas framework (Uhlén et al. 2015).

### Relationship Group Definition

Figure 7 groups were defined a priori from snoRNA detection, genomic context, validated target biotype, host–gene class, and prior evidence of interaction with the host transcript. We started by determining snoRNAs functionally coupled with their host gene, which we defined as including rRNA-targeting snoRNAs hosted by a ribosomal protein, ribosome biogenesis, or translation gene, and snRNA-targeting snoRNAs hosted by an RNA-processing gene. Among this group of snoRNAs, a subset were reported to interact with their own host transcript (Table S1 in Bergeron et al. 2023a). The snoRNAs attributed to the Bidirectional relation group are those displaying both the capacity to regulate their host gene and a functional coupling with their host gene, representing the pairs of snoRNAs and host genes with the strongest relationships. SnoRNAs functionally coupled with their host gene, but with no evidence of regulation of the maturation of their host gene, were attributed to the Functional coupling group. The remaining expressed, hosted snoRNAs were assigned to the Passenger relationship group. The other background group comprised intergenic snoRNAs and snoRNAs not detected in the analyzed tissues. These operational groups support a descriptive comparison and conceptual synthesis; they are not an unsupervised classification.

### Orthology Heatmap Filtering and Visualization

For the Fig. 5a visualization, we retained 20 well-annotated vertebrates spanning major lineages: chimpanzee, gorilla, macaque, rabbit, mouse, rat, dog, horse, cattle, pig, African elephant, opossum, platypus, chicken, zebra finch, anole, Xenopus, coelacanth, zebrafish, and medaka. The panel includes the 459 expressed human snoRNAs with at least one retained ortholog. Quantitative host-context summaries used all species with sufficient annotation and ortholog representation; species with orthologs for fewer than 10% of these human snoRNAs were excluded.

Heatmaps were displayed without hierarchical clustering. Rows were ordered by human host–gene class, decreasing number of represented orthologs among the selected species, and then decreasing same-host-gene retention; species were arranged in broad phylogenetic order from primates to teleost fishes.

### Statistical Analysis

Statistical analyses were performed using Python (v3.12.12; with the scipy (v1.16.1) library.

Continuous distributions were compared with 2-sided Mann–Whitney *U* tests. Host-class representation was summarized as fold enrichment (host proportion divided by background proportion) and tested with 2-sided Fisher exact tests. One-sided Fisher exact tests were used for enrichment of hosts carrying 4 or more expressed snoRNAs (Fig. 1f) and for target-category enrichment (Fig. 1g). Sample sizes are reported in the corresponding figures or tables.

Multiple testing correction was performed using the Benjamini–Hochberg procedure to control the false discovery rate.

Only the highest statistical significance is shown by brackets where applicable. Asterisks denote adjusted *P* < 0.05 (*), *P* < 0.01 (**), and *P* < 0.001 (***). Exact adjusted *P*-values are provided in the supplementary tables.

### Software and Computational Environment

Analyses were conducted in Python v3.12.12 using:

- pandas (v2.2.3)
- numpy (v2.1.2)
- scipy (v1.16.1)
- seaborn (v0.13.2)
- matplotlib (v3.9.2)

## Supplementary Material

evag269_Supplementary_Data

## Data Availability

See supplemental material for human snoRNA annotations used and *P*-values. The abundance determination pipeline is available at https://github.com/scottgroup/snorna_exprof.git. The host gene classification script is available at https://github.com/scottgroup/Classification_Genes.
