# Supplementary material for: SnoRNA Evolution, Genomic Context, and Mobility: The Role of Function and Host Gene Association
Source: Genome Biol Evol. 2026 Oct 24;18(11):evag269. doi: 10.1093/gbe/evag269 (PMC13736004; doi:10.1093/gbe/evag269)
Supplement: Supplementary file 1 [file evag269_supplementary_data.zip › All_Supplemental.pdf]

SnoRNA Evolution, Genomic Context and Mobility: The Role of Function and Host Gene Association

Supplemental Figures

Samuel Coderre[1,2]*, Alphonse Birane Thiaw[1,2], Michelle S Scott[1,2]

[1] Département de biochimie et de génomique fonctionnelle, Faculté de médecine et des sciences de la santé, Université de Sherbrooke, Sherbrooke, Québec, J1E 4K8, Canada

[2] Centre de recherche du Centre hospitalier universitaire de Sherbrooke (CRCHUS), Sherbrooke, Québec, J1H 5N3, Canada

[*] To whom correspondence should be addressed at : michelle.scott@usherbrooke.ca

Figure S1 Host classification support

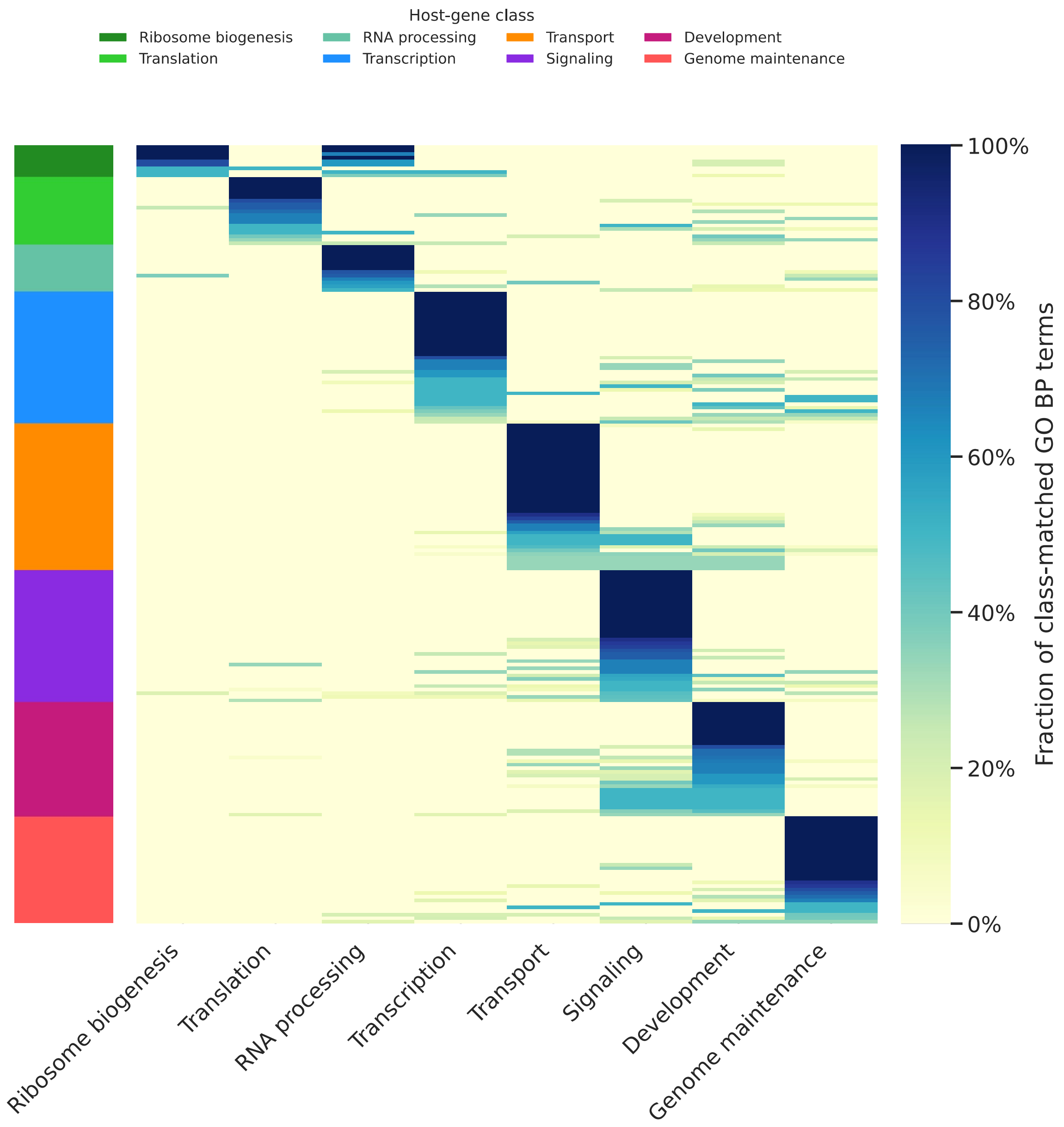

Biological-process (BP) support for 218 host genes across eight GO-based classes. Each row is a human host gene; each column gives the fraction of informative BP terms supporting that class. Fractions are non-exclusive because one term can support several classes. The left bar gives the assigned class. Ribosomal protein, lncRNA and other-coding hosts are excluded. Median assigned-class support is 0.95 (interquartile range 0.55–1.00). Unambiguous BP attribution requires strictly greater support for the assigned class than for every other class: 178/218 genes (81.7%) meet this criterion.

Figure S2 SnoRNA abundance by tissue

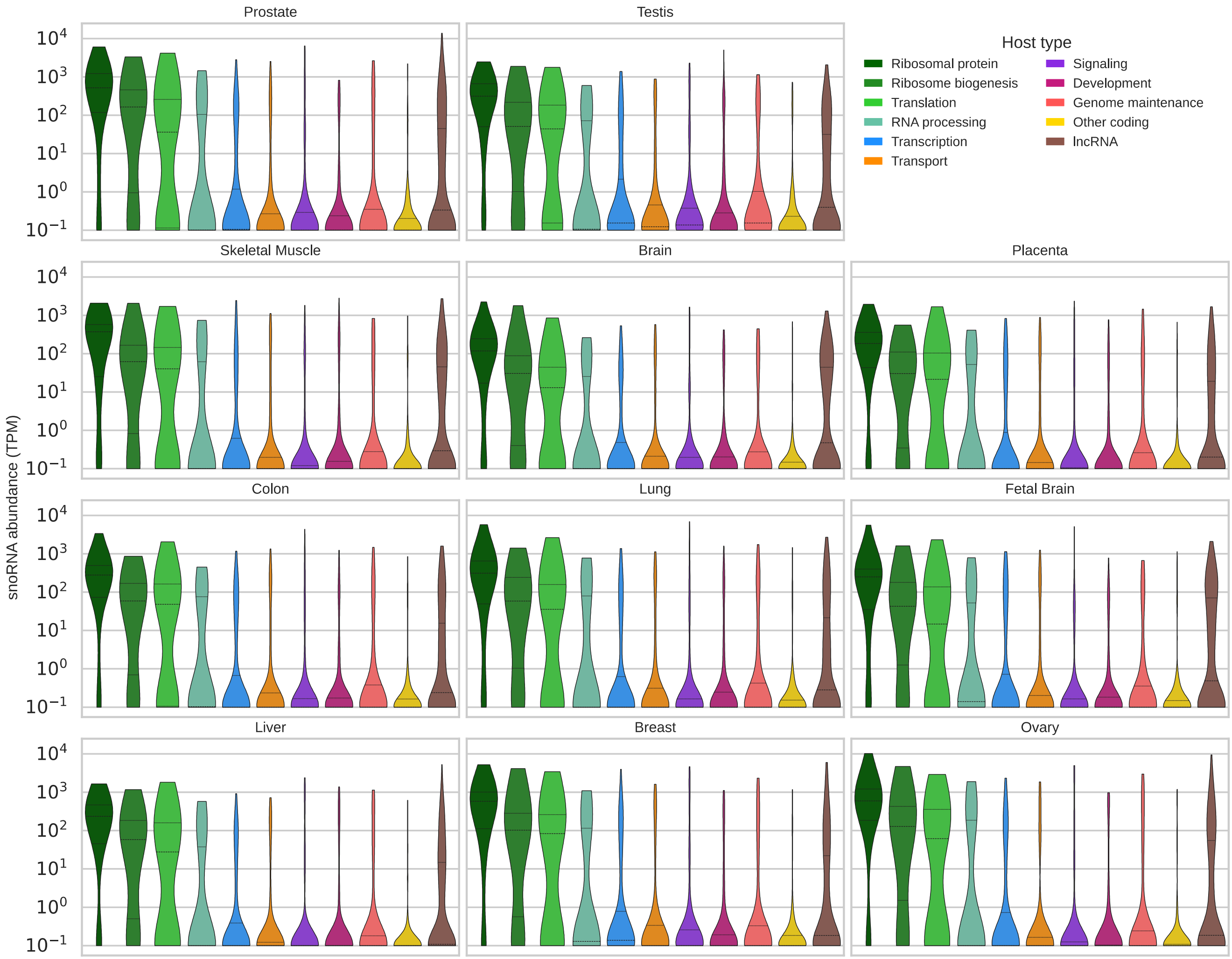

Violin plots of snoRNA abundance (TPM) in 11 human tissues (prostate, testis, skeletal muscle, brain, placenta, colon, lung, fetal brain, liver, breast and ovary), grouped by host-gene class. Each panel shows one tissue. The abundance axis is logarithmic (base 10). Densities were estimated on log10-transformed TPM values. Values below 0.1 TPM were plotted at 0.1 TPM.

Figure S3 SnoRNA abundance by host class

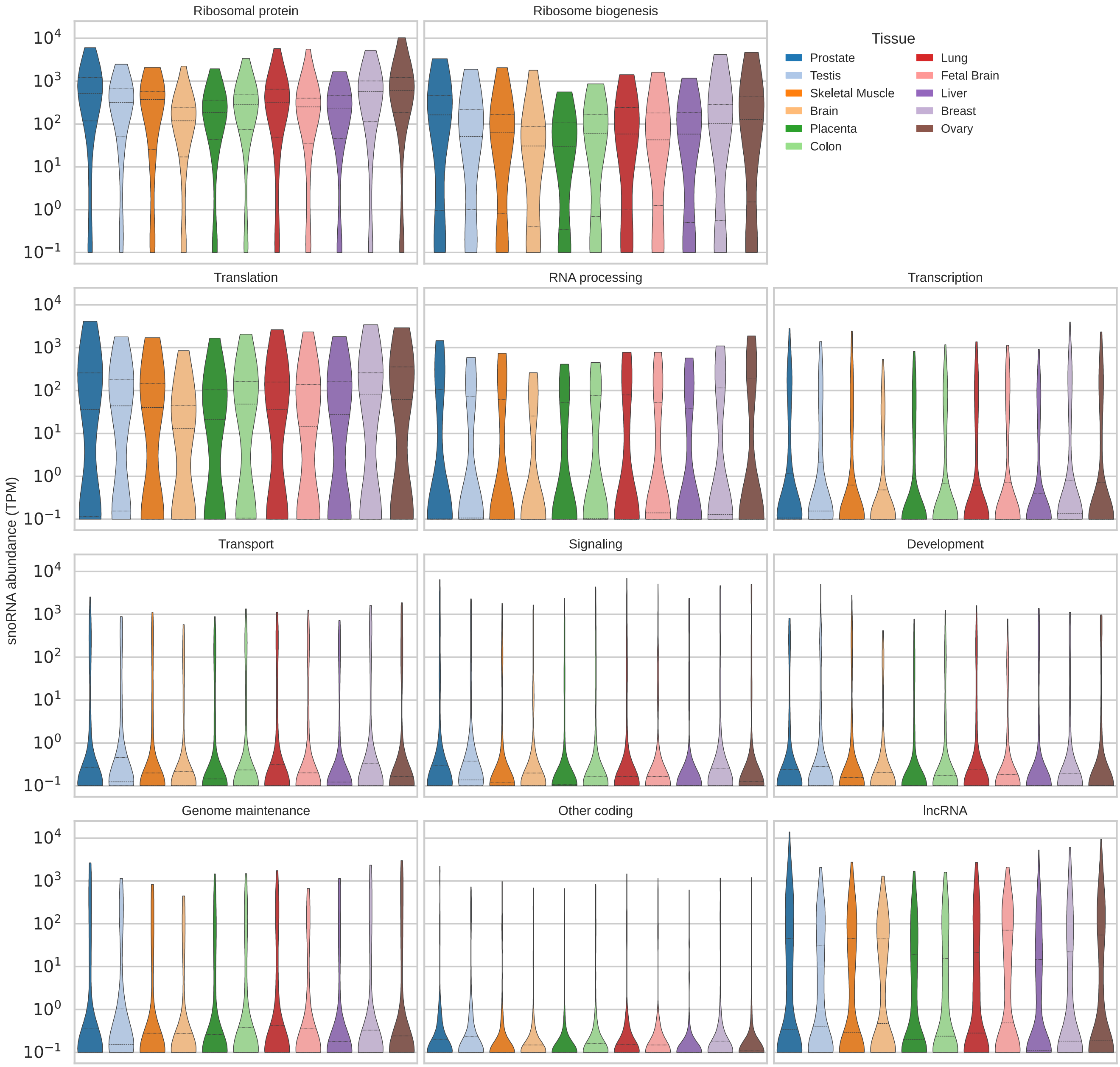

Violin plots of snoRNA abundance (TPM) across 11 human tissues, grouped by host-gene class. Each panel shows one host-gene class, and colors identify tissues. The abundance axis is logarithmic (base 10). Densities were estimated on log10-transformed TPM values. Values below 0.1 TPM were plotted at 0.1 TPM.

Figure S4 SnoRNA to host abundance ratios

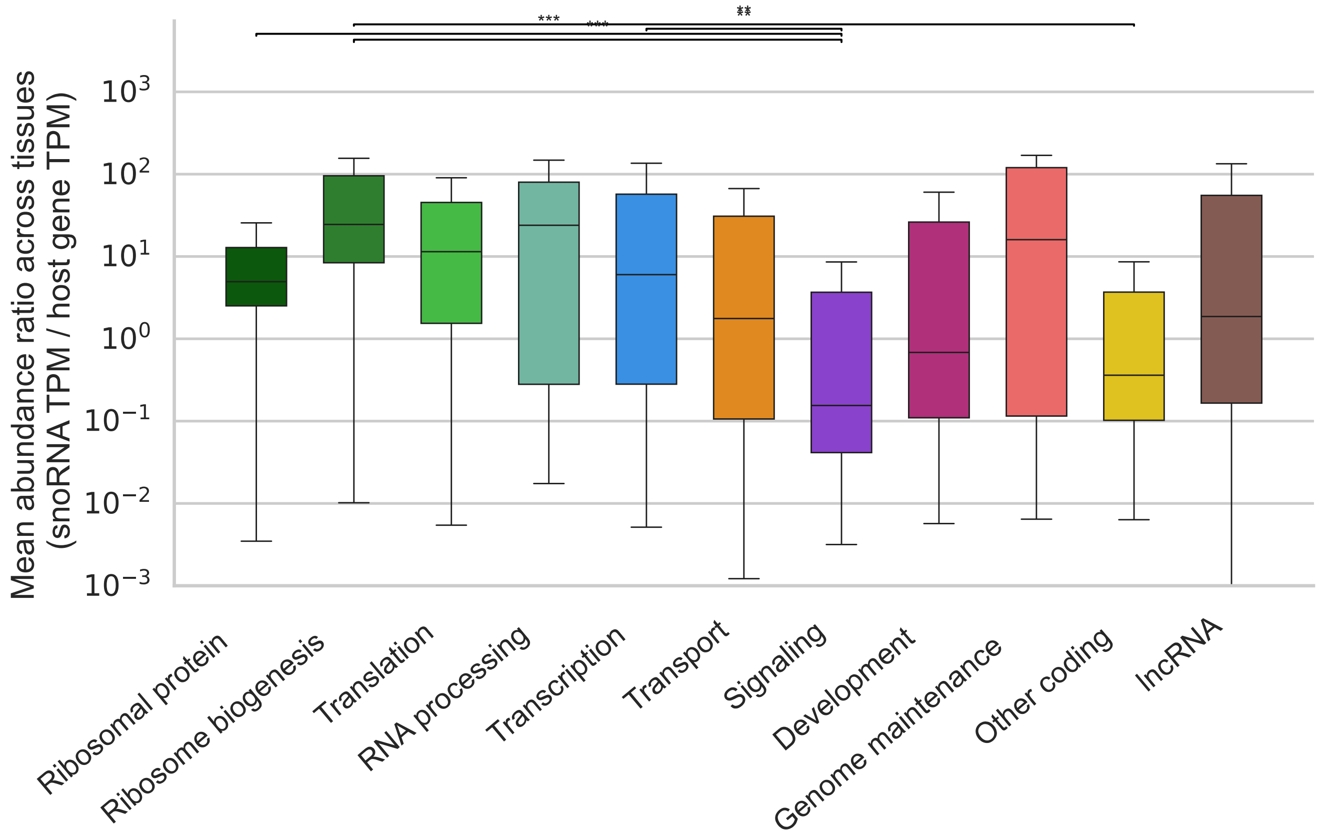

Distribution of snoRNA-to-host-transcript abundance ratios, grouped by host-gene class. Each observation is the mean of the tissue-specific snoRNA-to-host-transcript TPM ratios across the analyzed tissues. The abundance-ratio axis is logarithmic (base 10). These ratios describe steady-state RNA abundance, not transcription rates. Brackets indicate two-sided Mann–Whitney U comparisons with Benjamini–Hochberg correction; * adjusted $p < 0.05$, ** adjusted $p < 0.01$ and *** adjusted $p < 0.001$.

Figure S5 SnoRNA conservation by expression pattern

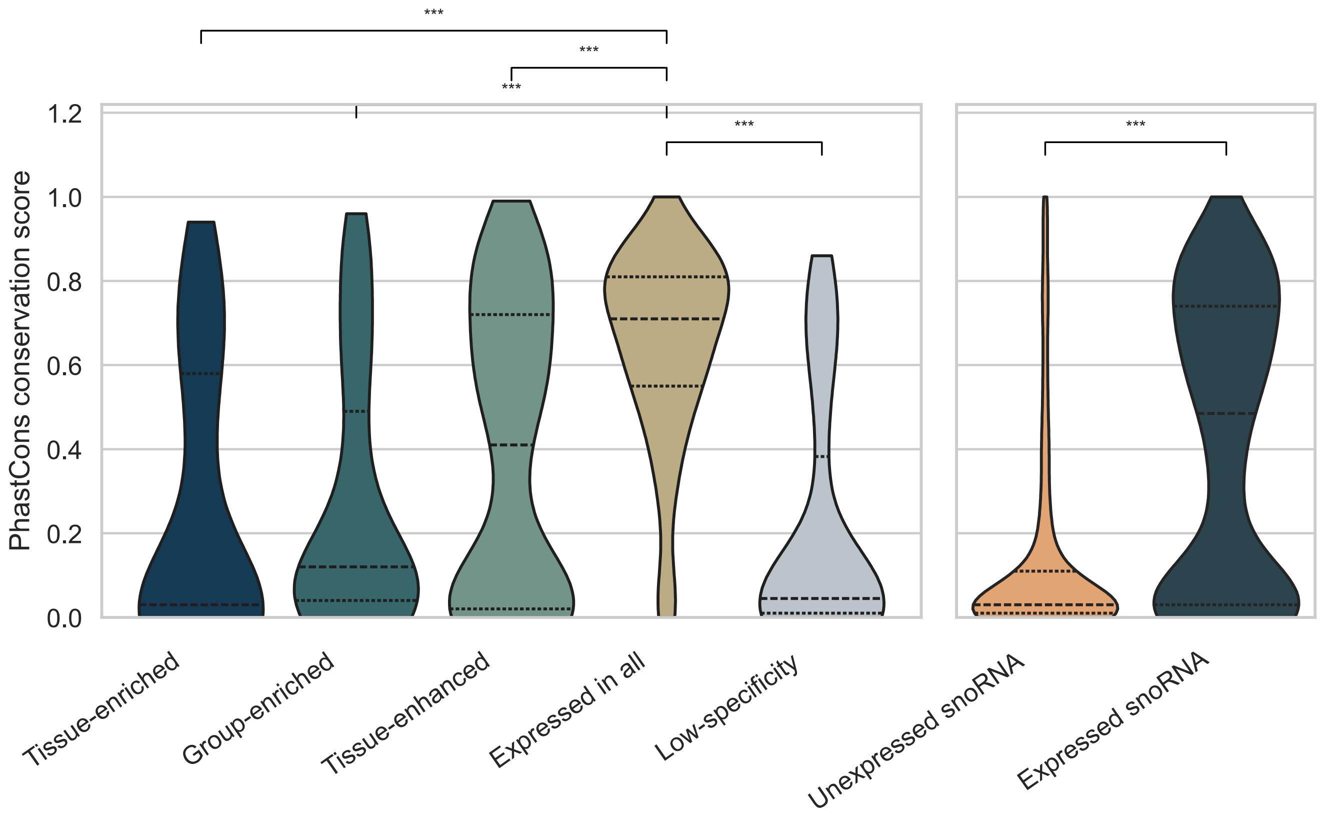

Distribution of PhastCons sequence-conservation scores for human snoRNAs grouped by their tissue-expression pattern. The left panel shows tissue-specificity categories defined using the Human Protein Atlas framework and the abundance profiles across the analyzed human tissues. The right panel compares undetected and expressed snoRNAs. Brackets indicate two-sided Mann–Whitney U comparisons with Benjamini–Hochberg correction; * adjusted $p < 0.05$, ** adjusted $p < 0.01$ and *** adjusted $p < 0.001$.

Figure S6 Host classes of snoRNA families

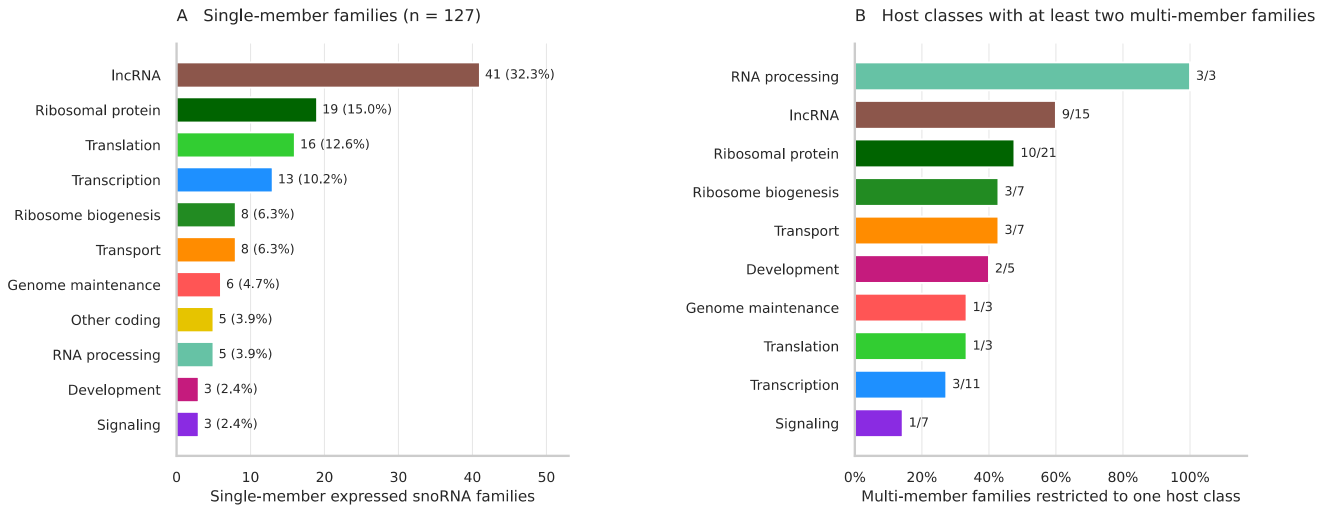

Host-class distribution of expressed human snoRNA families. (A) Counts and percentages for families with a single expressed human member. The panel displays 127 families after omission of the one intergenic family; percentages use the 127 displayed families as the denominator. The manuscript total of 128 includes that intergenic family. (B) Fraction of multi-member families restricted to one host-gene class, grouped by their dominant host class. Only the ten host classes represented by at least two multi-member families are displayed (82 families in total). Labels give the number restricted to a single class over the total number in that class.

Figure S7 SnoRNA and host ortholog counts

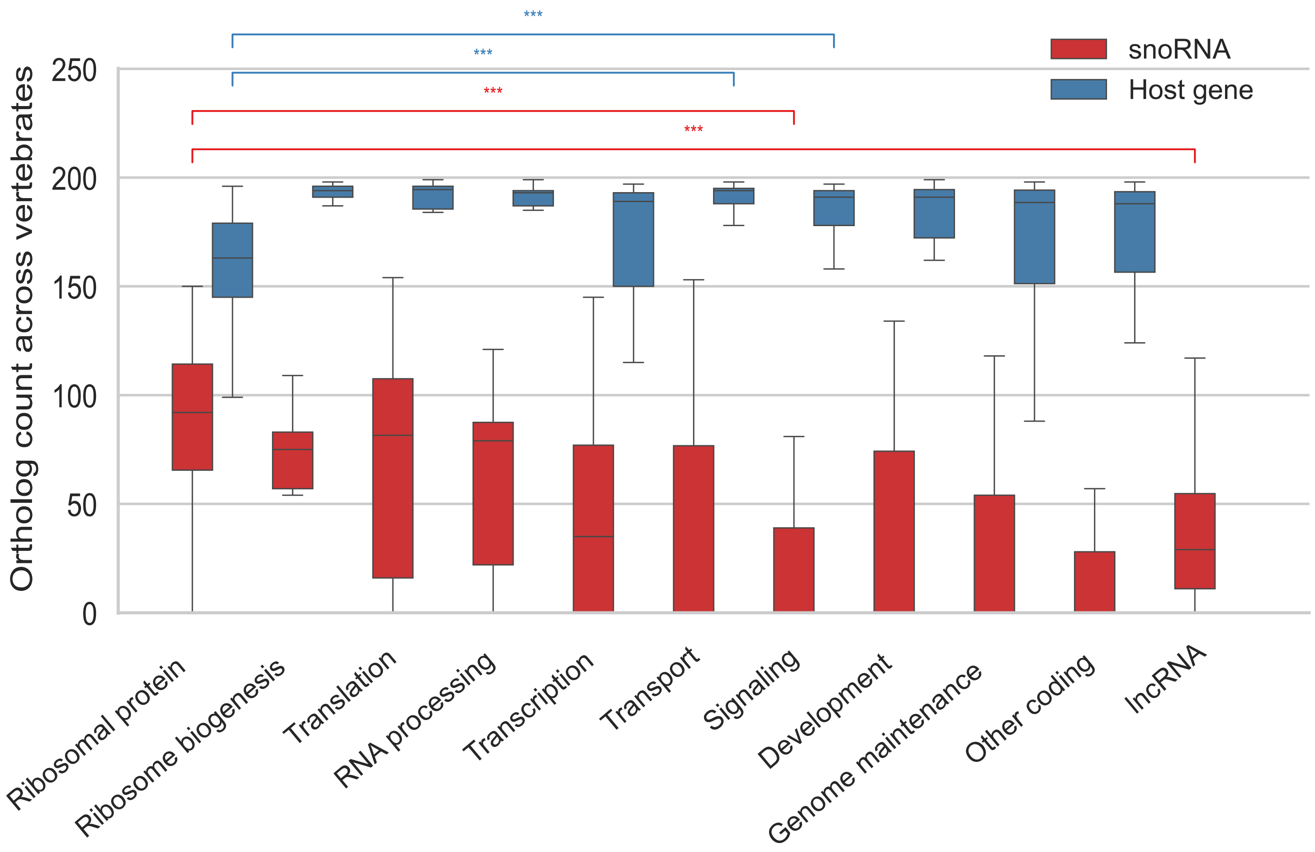

Distribution of ortholog counts for expressed human snoRNAs and their host genes across 197 vertebrate genomes, grouped by the human host-gene class. Red boxes represent snoRNAs and blue boxes their host genes. No orthologs of the human lncRNA hosts were retrieved in this dataset; the zero counts reflect the available orthology annotations and should not be interpreted as evidence that these loci are biologically absent from other vertebrates. Brackets indicate two-sided Mann–Whitney U comparisons between host classes, with Benjamini–Hochberg correction separately for snoRNAs and host genes; * adjusted $p < 0.05$, ** adjusted $p < 0.01$ and *** adjusted $p < 0.001$.

Figure S8 Flanking exon conservation

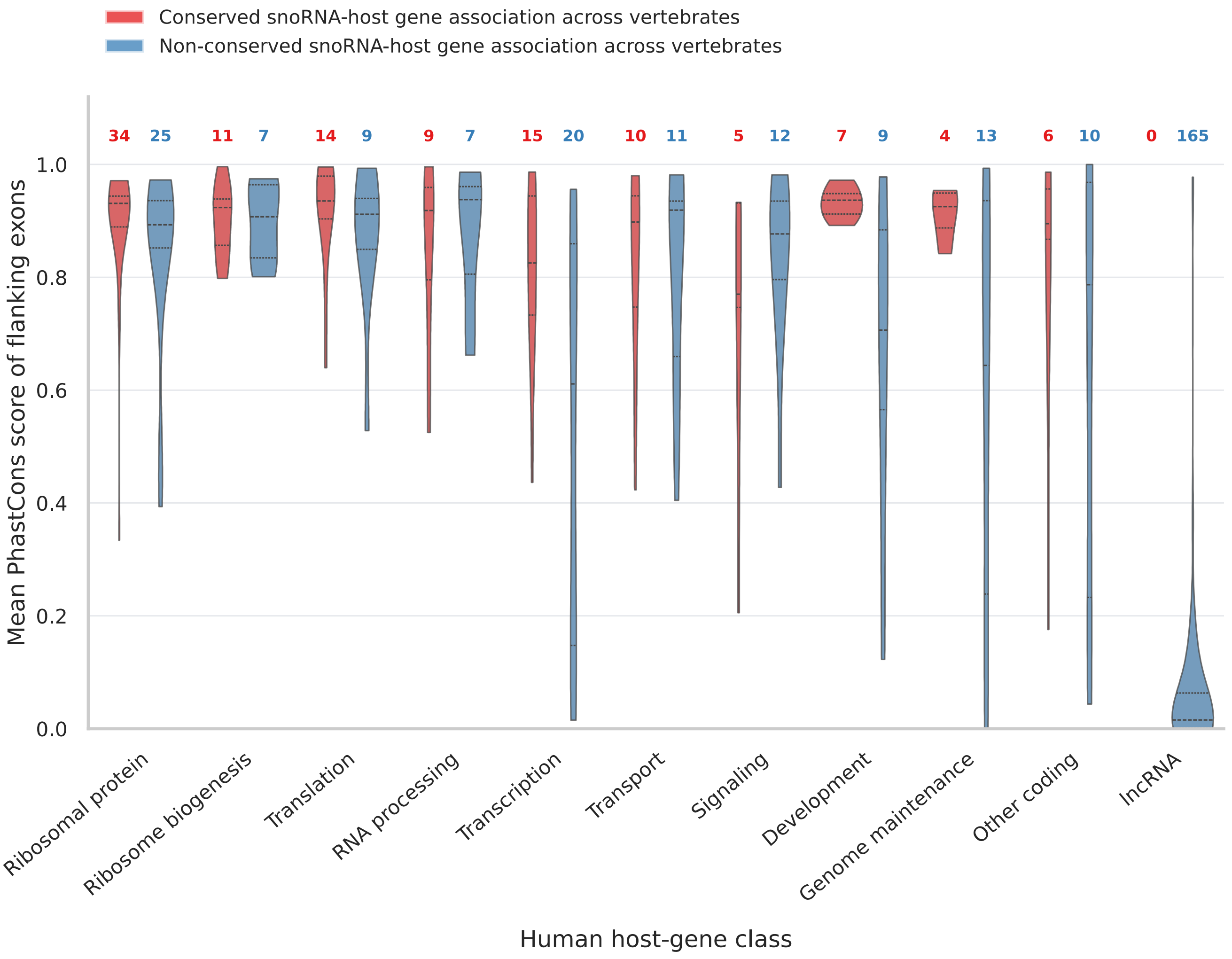

Mean 100-way PhastCons scores of up to 500 nt of each exon flanking snoRNA-containing introns, grouped by human host-gene class. Conserved snoRNA-host gene associations (red, n = 115) retain the human host gene in >50% of available orthologs; non-conserved associations (blue, n = 288) comprise the remaining pairings with at least one ortholog. All available orthologs across 196 species were considered. Violins show quartiles; numbers indicate sample sizes. No within-class difference was significant after two-sided Mann–Whitney U tests with Benjamini–Hochberg correction.
